# Supplementary material for: Chromosome-level assemblies of multiple Arabidopsis genomes reveal hotspots of rearrangements with altered evolutionary dynamics
Source: Nat Commun. 2020 Feb 20;11:989. doi: 10.1038/s41467-020-14779-y (PMC7033125; doi:10.1038/s41467-020-14779-y)
Supplement: Supplementary file 1 — Supplementary Information [file 41467_2020_14779_MOESM1_ESM.pdf]

Chromosome-level assemblies of multiple *Arabidopsis* genomes reveal hotspots of rearrangements with altered evolutionary dynamics

Jiao *et al.*

Supplementary Table 1. Whole genome DNA sequencing data.

Accession		PacBio			Illumina paired-end		
	Stock number	Reads	Mean Length	Depth	Pairs	Length	Depth
An-1	N944	1,419,095	6,745	70	52,408,462	101	78.4
C24	CS906	1,026,041	6,011	45	37,543,070	101	56.2
Cvi-0	N8580	1,358,030	7,132	71	44,119,747	101	66.0
Eri-1	CS22548	1,364,672	6,402	64	37,489,627	101	56.1
L*er*	N20	1,312,624	7,140	69	48,030,713	101	71.9
Kyo	JW10231	1,254,045	6,599	61	42,068,024	101	62.9
Sha	CS929	853,955	8,281	52	50,271,422	101	75.2

Supplementary Table 2. Contig assembly statistics.

	An-1	C24	Cvi-0	Eri-1	Kyo	L*er*	Sha
**Contigs**	151	167	140	200	230	149	143
**Total bases**	120,109,838	119,191,266	119,730,512	120,766,191	122,179,079	120,316,059	120,265,401
**N25**	11,780,999	9,748,094	9,283,899	8,805,940	11,196,314	13,234,969	12,200,080
**N50**	8,166,377	4,802,170	7,363,394	4,757,538	9,149,088	11,168,341	6,996,577
**N75**	4,989,922	2,108,767	5,593,362	1,771,097	3,917,708	6,074,025	2,595,543
**N90**	1,333,704	593,800	1,975,628	657,186	949,167	804,247	1,088,930
**L25**	3	3	3	3	3	3	3
**L50**	6	8	7	8	6	5	6
**L75**	10	18	11	18	10	9	12
**L90**	17	34	17	34	19	18	23
**CN50**	8,166,377	4,802,170	6,994,145	4,757,538	9,149,088	13,234,969	12,200,080
**CL50**	2	2	2	2	2	1	1
**NG25**	11,780,999	8,058,848	9,238,557	7,925,394	11,196,314	13,234,969	12,200,080
**NG50**	7,186,630	3,510,498	6,994,145	4,279,918	9,149,088	8,759,918	5,319,415
**NG75**	2,765,233	1,004,113	2,403,042	1,106,604	2,177,877	2,307,265	1,494,253
**NG90**	0	0	0	0	12,670	0	0
**LG25**	3	4	4	4	3	3	3
**LG50**	7	10	8	10	6	6	7
**LG75**	13	26	15	25	13	12	18
**LG90**	0	0	0	0	132	0	0
**Min**	508	86	488	561	474	184	1,016
**Max**	14,839,891	10,639,543	12,641,456	12,399,038	14,879,663	14,816,562	14,310,783

Supplementary Table 3. Assembly evaluation of collapsed regions.

Accession	Number of collapsed regions	Cumulative size (bp)
An-1	288	187,700
C24	178	106,500
Col-0	326	508,800
Cvi-0	209	211,300
Eri-1	144	99,200
Kyo	187	115,200
L*er*	294	260,500
Sha	485	507,800

Supplementary Table 4. Assembly validation using genetic maps.

Cross	Assembly tested	Data	Markers	Aligned	Conflicts
Cvi-0 x Col-0	Cvi-0	Simon *et al.* [1]	94	93	0
L*er* x Col-0	L*er*	Singer *et al.* [2]	676	415	3
L*er* x Col-0	L*er*	Giraut *et al.* [3]	386	357	3

Note: Three genetic maps were used to validate the assemblies of Cvi-0 and L*er*. Evaluation of the conflicting markers revealed that all them of were likely caused by structural variations. Each of the conflicting markers was found on contigs which were supported by non-conflicting markers as well.

Supplementary Table 5. Location of rDNA clusters.

Accession	Chr	Start	End	Size	Unit Count
An-1	chr1	16,247,697	16,249,746	2,049	5
An-1	chr2	15,160	30,883	15,723	4
An-1	chr3	13,660,309	13,693,016	32,707	44
An-1	chr4	303	12,836	12,533	3
An-1	chr5	11,704,997	11,712,070	7,073	12
An-1	chr5	12,502,040	12,517,915	15,875	41
An-1	chr5	12,528,355	12,548,970	20,615	56
C24	chr1	15,480,421	15,481,987	1,566	4
C24	chr2	5,277	9,979	4,702	3
C24	chr3	14,477,578	14,508,366	30,788	40
C24	chr4	3,024,558	3,029,149	4,591	10
C24	chr5	11,167,024	11,173,642	6,618	11
C24	chr5	12,047,791	12,084,720	36,929	86
Cvi-0	chr1	15,291,551	15,293,036	1,485	1
Cvi-0	chr1	16,201,388	16,203,919	2,531	6
Cvi-0	chr3	13,553,363	13,557,056	3,693	8
Cvi-0	chr3	13,632,106	13,672,892	40,786	50
Cvi-0	chr3	13,693,322	13,720,728	27,406	49
Cvi-0	chr3	14,005,932	14,048,548	42,616	37
Cvi-0	chr4	3,078,679	3,107,071	28,392	57
Cvi-0	chr5	11,309,985	11,316,573	6,588	11
Eri-1	chr1	15,214,182	15,216,702	2,520	6
Eri-1	chr3	14,398,975	14,411,194	12,219	25
Eri-1	chr3	14,944,897	14,945,898	1,001	1
Eri-1	chr5	12,503,702	12,518,335	14,633	37
Eri-1	chr5	12,528,934	12,547,045	18,111	48
Kyo	chr1	15,615,850	15,617,896	2,046	5
Kyo	chr3	14,537,056	14,550,096	13,040	9
Kyo	chr3	15,246,489	15,247,490	1,001	1
Kyo	chr5	11,290,583	11,292,705	2,122	5
Kyo	chr5	11,328,618	11,335,208	6,590	11
Kyo	chr5	12,294,721	12,301,528	6,807	15
Kyo	chr5	12,324,038	12,325,865	1,827	4
L*er*	chr1	16,112,353	16,114,394	2,041	5
L*er*	chr2	24,660	34,941	10,281	2
L*er*	chr3	6,075,317	6,076,940	1,623	4
L*er*	chr3	14,214,467	14,253,261	38,794	7
L*er*	chr3	14,267,148	14,295,446	28,298	39
L*er*	chr4	4,789	31,377	26,588	6
L*er*	chr4	3,461,906	3,478,278	16,372	31
L*er*	chr5	11,240,780	11,255,952	15,172	31
L*er*	chr5	11,317,853	11,324,980	7,127	12
L*er*	chr5	12,403,488	12,410,158	6,670	18
L*er*	chr5	12,432,957	12,436,284	3,327	7
Sha	chr1	16,317,928	16,319,976	2,048	5
Sha	chr2	4,747	17,222	12,475	3
Sha	chr3	13,328,855	13,413,311	84,456	37
Sha	chr4	1,132	27,909	26,777	5
Sha	chr4	39,104	50,775	11,671	5
Sha	chr4	2,996,768	3,020,817	24,049	48
Sha	chr5	11,301,764	11,362,233	60,469	121
Sha	chr5	12,959,621	12,973,107	13,486	34

Supplementary Table 6. Sequence annotation near assembly gaps.

Assembly	Gaps	Pericentromere	rDNA	TE	All
An-1	40	26	2	38	95.00%
C24	68	34	3	59	86.76%
Cvi-0	38	25	6	35	92.11%
Eri-1	58	22	2	49	84.48%
Kyo	46	31	3	41	89.13%
L*er*	44	27	4	41	93.18%
Sha	49	30	5	47	97.96%

Note: Assembly gaps can reside in the pericentromere, in or close to ribosomal DNA cluster (distance to the closest rDNA cluster < 5kb) or in or close to transposable elements (distance to closest TE < 500bp). As some of them may simultaneously locate in the pericentromere and rDNA clusters or TEs, the last column indicates the percent of gaps that matched at least one of these features.

Supplementary Table 7. Assembly evaluation.

Accession	Genes without any blastn hit (fully deleted)	Genes without high quality blastn hit (partially deleted)	Percent of genes with good blastn hit	No. of genes without full (<60%) short read coverage
**An-1**	112	75	99.3%	92.5%
**C24**	120	88	99.2%	85.6%
**Cvi-0**	136	95	99.2%	87.4%
**Eri-1**	102	76	99.4%	88.2%
**Kyo**	85	79	99.4%	87.8%
**L*er***	107	74	99.3%	89.0%
**Sha**	159	89	99.1%	75.8%

Note: To check the assembly completeness, we aligned all 27,445 reference genes against each assembly using blastn**.** Genes without any blastn hit are fully absent in the assembly (second column). Genes without high-quality blastn hit are only partially present in the assembly (third column). ("High-quality blastn hits" were defined as blastn hits with identity larger than 85 and coverage larger than 95%.)

In order to check if the genes that are absent in the assemblies were just not assembled or were truly missing in the genomes, we further aligned the Illumina whole-genome reads from each accession against Col-0 reference sequence. The last column indicates how many of the genes without blastn hit or good blastn hit have short read alignment coverage in less than 60% of their sequence (and are thus mostly like truly absent in the sequenced genomes).

Supplementary Table 8. Gene and repeat annotations.

	Col-0	An-1	C24	Cvi-0	Eri-1	Kyo	L*er*	Sha
**Protein-coding genes (Araport11)**	27,445	27,342	27,214	27,098	27,285	27,574	27,376	27,293
**miRNAs**	298	316	301	297	301	313	296	301
**snRNAs**	75	76	78	76	77	77	78	75
**snoRNAs**	551	602	529	553	571	578	562	580
**rRNAs**	550	501	347	661	387	688	395	1,011
**tRNAs**	661	564	545	585	589	561	571	511
**other RNAs**	75	41	43	48	48	43	41	26
**DNA transposons**	10,278	10,095	10,452	10,769	10,834	11,265	10,689	10,702
**retrotransposons**	8,598	8,797	8,493	8,724	8,601	8,790	8,570	9,072
**DNA transposon content**	4.96%	4.79%	4.77%	4.81%	4.99%	4.99%	4.81%	4.78%
**retrotransposon content**	8.28%	8.86%	8.40%	8.23%	8.81%	8.62%	8.50%	8.56%
**Complete repeat content**	17.55%	17.94%	17.42%	17.47%	18.28%	18.57%	17.93%	18.42%

Note: The non-coding RNA and repeat annotation were predicted with Infernal and RepeatMasker, respectively.

Supplementary Table 9. Number and total length of rearranged regions.

Accession	INV	ITX	CTX	DUP-Loss	RefSp.	DUP-Gain	AccSp.
Number							
An-1	33	364	365	2,181	5,551	2,107	5,805
C24	37	473	508	2,423	6,305	2,207	6,455
Cvi-0	37	566	626	2,546	7,478	2,604	7,705
Eri-1	42	410	462	2,134	5,718	2,057	6,293
Kyo	33	368	430	2,026	5,681	2,277	6,516
L*er*	46	509	485	2,409	6,085	2,470	6,509
Sha	37	460	487	2,347	6,391	2,631	6,837
Length							
An-1	1,824,867	985,071	862,756	3,861,135	5,320,292	3,377,683	5,171,246
C24	1,742,898	1,367,338	1,311,211	4,562,135	5,929,351	3,301,993	5,900,176
Cvi-0	1,485,583	1,657,606	1,273,284	4,417,754	6,345,126	4,074,912	6,294,286
Eri-1	1,709,695	975,394	1,016,615	4,063,236	5,405,177	3,323,824	5,730,514
Kyo	1,559,482	988,729	1,147,826	3,917,699	5,056,775	3,790,715	5,511,508
L*er*	1,799,626	1,402,826	1,102,662	4,284,186	5,600,844	4,005,952	5,768,803
Sha	4,214,442	1,254,696	1,181,706	4,408,231	6,526,286	4,291,496	6,018,238

Note: INV: inversion; ITX: intra-chromosome translocation; CTX: inter-chromosome translocation; DUP-Loss: duplication loss in accession (i.e.: the reference Col-0 has more copies); DUP-Gain: duplication gain in accession (i.e.: the accession has more copies); RefSp: regions specific to the reference genome; AccSp: regions specific to the accession.

Supplementary Table 10. The percent of genomic rearrangements which reside in or overlap with pericentromeric regions.

Genome	INV	ITX	CTX	DUP-Loss	DUP-Gain	Total
An-1	86.2%	47.4%	41.8%	52.3%	56.6%	52.7%
C24	76.0%	49.5%	45.4%	56.7%	56.3%	54.8%
Cvi-0	89.3%	51.8%	38.4%	52.4%	56.1%	52.2%
Eri-1	85.0%	48.9%	45.6%	55.3%	55.5%	53.7%
Kyo	86.6%	50.0%	47.6%	52.3%	55.9%	53.1%
L*er*	82.5%	59.0%	48.1%	59.6%	62.2%	59.4%
Sha	61.5%	52.7%	41.4%	54.8%	61.3%	56.1%
total	45.3%	51.6%	43.8%	54.8%	57.8%	54.6%

Note: INV: inversion; ITX: intra-chromosome translocation; CTX: inter-chromosome translocation; DUP-Loss: duplication in reference but not in the accession genome; DUP-Gain: duplication in the accession genome but not in the reference genome.

Supplementary Table 11. Inversions larger than 50kb.

Accession	Chr	Start	End	Reference Length	Chr	Start	End	Query Length
An-1	Chr1	14,501,429	15,074,096	572,667	Chr1	14,867,422	15,215,011	347,589
Sha	Chr1	17,660,763	17,879,638	218,875	Chr1	17,552,872	17,764,136	211,264
C24	Chr1	23,188,572	23,504,543	315,971	Chr1	22,126,192	22,444,978	318,786
L*er*	Chr2	12,661,878	12,731,490	69,612	Chr2	12,361,365	12,433,285	71,920
Sha	Chr3	2,774,870	5,252,859	2,477,989	Chr3	2,798,243	5,282,580	2,484,337
L*er*	Chr3	8,295,153	8,465,270	170,117	Chr3	8,351,497	8,521,688	170,191
An-1	Chr3	12,482,569	12,618,513	135,944	Chr3	12,419,542	12,562,554	143,012
An-1	Chr3	13,602,813	13,921,313	318,500	Chr3	13,323,011	13,379,922	56,911
C24	Chr3	13,602,813	13,924,434	321,621	Chr3	14,225,825	14,285,314	59,489
Eri-1	Chr3	13,602,813	13,923,880	321,067	Chr3	14,090,720	14,163,036	72,316
Kyo	Chr3	13,602,813	13,921,313	318,500	Chr3	14,288,380	14,342,210	53,830
Sha	Chr3	14,336,990	14,554,838	217,848	Chr3	13,546,260	13,785,045	238,785
An-1	Chr4	1,612,606	2,782,625	1,170,019	Chr4	1,652,389	2,880,783	1,228,394
Cvi-0	Chr4	1,612,606	2,782,625	1,170,019	Chr4	1,584,780	2,849,231	1,264,451
Eri-1	Chr4	1,612,606	2,782,625	1,170,019	Chr4	1,690,889	2,870,416	1,179,527
Kyo	Chr4	1,612,606	2,782,625	1,170,019	Chr4	1,666,349	2,828,956	1,162,607
L*er*	Chr4	1,612,606	2,782,625	1,170,019	Chr4	1,746,529	2,898,561	1,152,032
Sha	Chr4	1,612,606	2,782,625	1,170,019	Chr4	1,655,631	2,860,573	1,204,942
C24	Chr4	1,771,815	2,782,625	1,010,810	Chr4	1,628,501	2,773,489	1,144,988
Sha	Chr4	4,356,088	4,459,766	103,678	Chr4	4,583,599	4,658,107	74,508
C24	Chr4	4,572,269	4,692,550	120,281	Chr4	5,054,623	5,231,295	176,672
Eri-1	Chr4	4,572,269	4,684,953	112,684	Chr4	4,301,368	4,383,248	81,880
Kyo	Chr4	4,573,080	4,692,550	119,470	Chr4	4,533,759	4,689,570	155,811
Sha	Chr5	11,730,380	11,918,318	187,938	Chr5	11,786,872	11,986,397	199,525
An-1	Chr5	11,806,527	12,002,521	195,994	Chr5	11,646,437	11,775,074	128,637
An-1	Chr5	12,437,159	13,049,679	612,520	Chr5	12,282,399	12,909,890	627,491
Eri-1	Chr5	12,437,159	13,049,679	612,520	Chr5	12,323,868	12,905,576	581,708
Kyo	Chr5	12,437,159	12,989,685	552,526	Chr5	12,188,346	12,650,586	462,240
Sha	Chr5	12,437,159	12,989,685	552,526	Chr5	12,846,278	13,225,872	379,594
C24	Chr5	12,477,370	13,049,679	572,309	Chr5	11,887,516	12,455,714	568,198
Cvi-0	Chr5	12,539,575	13,049,679	510,104	Chr5	12,289,995	12,635,489	345,494
L*er*	Chr5	12,592,489	12,989,685	397,196	Chr5	12,316,710	12,555,454	238,744
L*er*	Chr5	13,050,974	13,284,321	233,347	Chr5	12,555,453	12,810,352	254,899

Supplementary Table 12. Number and total length of local sequence variation in syntenic and rearranged regions.

Accession	Region	SNP	S-Indel	L-Indel	HDR	CPG	CPL
Number							
An-1	SYN	637,248	150,172	1,372	1,582	400	400
An-1	GR	81,377	9,591	70	48	58	41
C24	SYN	720,846	168,230	1,604	1,690	464	441
C24	GR	103,207	12,332	95	62	60	56
Cvi-0	SYN	892,125	209,003	1,851	2,282	522	541
Cvi-0	GR	114,058	13,430	95	83	61	56
Eri-1	SYN	682,249	159,143	1,438	1,582	441	439
Eri-1	GR	84,278	9,696	73	53	55	50
Kyo	SYN	691,263	159,418	1,499	1,667	433	459
Kyo	GR	81,309	9,896	77	58	48	52
L*er*	SYN	693,354	161,088	1,439	1,687	447	467
L*er*	GR	102,048	11,952	83	56	52	53
Sha	SYN	727,954	170,344	1,533	1,793	465	491
Sha	GR	114,816	15,077	108	82	66	70
Length							
An-1	SYN	637,248	532,662	538,146	953,561	367,053	341,470
An-1	GR	81,377	33,006	26,918	26,336	158,342	106,342
C24	SYN	720,846	602,294	653,268	1,081,456	463,818	403,270
C24	GR	103,207	41,370	20,938	44,956	288,835	150,235
Cvi-0	SYN	892,125	742,298	723,616	1,556,755	551,227	492,673
Cvi-0	GR	114,058	45,222	27,663	49,091	154,123	121,634
Eri-1	SYN	682,249	570,014	566,821	972,935	393,342	469,857
Eri-1	GR	84,278	32,141	27,734	27,278	139,160	209,105
Kyo	SYN	691,263	567,521	585,439	986,372	366,991	522,942
Kyo	GR	81,309	33,208	29,441	54,438	132,861	162,146
L*er*	SYN	693,354	571,606	558,989	946,557	489,357	481,803
L*er*	GR	102,048	40,617	22,175	38,796	120,753	224,427
Sha	SYN	727,954	609,894	606,149	1,162,736	475,067	533,052
Sha	GR	114,816	51,339	41,818	40,173	144,841	202,717

Note: SYN: syntenic regions; GR: rearranged regions including inversion, translocation and duplications; S-Indel: short indels; L-Indel: long indels; HDR: highly divergent regions; CPG: copy gain variation; CPL: copy-loss variation

Supplementary Table 13. Gene family contraction and expansion between Col-0 and each of the other seven genomes.

Acc.	NvN	1v2	2v1	1v3	3v1	2v3	3v2	Ref>Qry	Qry>Ref	ColSp
An-1	25,561	155	330	12	36	46	138	358	131	678
C24	25,228	185	402	13	81	72	168	450	147	699
Cvi-0	25,019	151	444	16	84	100	207	452	149	823
Eri-1	25,379	175	378	17	63	62	120	397	154	704
Kyo	25,556	168	358	19	45	56	147	381	102	613
L*er*	25,399	164	388	14	84	72	144	381	135	665
Sha	25,184	163	394	14	78	74	159	448	139	792

Note: Gene copy number variation between Col-0 and each of other accessions based on gene family clustering. Acc.: accessions; NvN: both the Col-0 and the query genomes have the same number of genes in the respective gene families. 1v2, 2v1, 1v3, 3v1, 2v3, 3v2 indicate the corresponding number of Col-0 genes and accession genes in a gene family. “Ref>Qry” and “Qry>Ref” refer to the number of Col-0 genes in cases where either the reference or a divergent accession has more genes (>3) in a particular gene family. ColSp: genes present in the reference Col-0 genome but absent in the accession genome.

Supplementary Table 14. Non-reference genes.

Acc.	Novel	Novel2	AccSp	*A. lyrata* ortholog	RNA-seq support
An-1	539	403	136	166	164
C24	590	406	184	198	236
Cvi-0	598	384	214	175	179
Eri-1	537	416	121	171	n/a
Kyo	533	411	122	177	n/a
L*er*	555	420	135	172	270
Sha	551	413	138	173	146
Total	1,941	891	1,050	452	

Note: The values in the “Total” row depict the numbers of gene families of the genes in the individual accessions (i.e. the non-redundant gene numbers). Novel2: genes shared by at least two accessions. AccSp: genes specific to an accession. n/a: no RNA-seq data available for these genomes.

Supplementary Table 15. R gene copy number differences in R gene clusters.

ID	Chr	Start	End	An-1	C24	Col-0	Cvi-0	Eri-1	Kyo	L*er*	Sha
RC001	Chr1	4,140,216	4,147,939	2	2	2	2	2	1	1	2
RC002	Chr1	4,174,875	4,182,593	2	2	2	2	2	2	2	2
RC003	Chr1	6,052,936	6,060,851	3	3	3	3	3	3	3	3
RC004	Chr1	9,433,393	9,446,223	2	2	2	2	2	1	2	2
RC005	Chr1	21,167,565	21,185,495	6	3	3	5	3	2	3	3
RC006	Chr1	21,345,445	21,359,872	3	4	3	3	3	2	3	3
RC007	Chr1	21,420,244	21,428,078	2	0	2	2	1	1	2	0
RC008	Chr1	21,690,642	21,704,982	3	2	3	2	2	2	3	2
RC009	Chr1	21,746,354	21,833,018	5	1	5	1	2	1	4	2
RC010	Chr1	22,551,271	22,560,757	3	3	2	1	2	3	2	1
RC011	Chr1	22,607,462	22,616,373	1	1	2	1	1	1	1	1
RC012	Chr1	23,494,643	23,502,321	0	2	2	0	2	0	2	0
RC013	Chr1	23,641,770	23,655,453	3	3	3	3	3	3	3	3
RC014	Chr1	23,701,408	23,716,206	3	3	3	4	3	4	4	4
RC015	Chr1	27,409,196	27,445,997	8	9	11	8	13	6	11	9
RC016	Chr2	7,410,704	7,427,119	3	3	3	3	3	3	3	3
RC017	Chr3	1,105,650	1,112,437	2	2	2	2	2	2	2	2
RC018	Chr3	4,851,990	4,861,293	2	2	2	0	2	2	2	2
RC019	Chr3	16,044,662	16,096,477	1	2	2	0	2	2	0	4
RC020	Chr3	16,195,848	16,221,722	12	7	2	5	4	4	8	2
RC021	Chr3	17,206,183	17,215,755	3	2	2	2	3	1	2	1
RC022	Chr3	19,121,687	19,130,705	2	2	2	2	2	2	2	2
RC023	Chr4	5,940,186	5,973,113	3	0	3	0	3	3	0	3
RC024	Chr4	6,811,103	6,899,130	2	2	2	2	2	2	2	2
RC025	Chr4	7,196,991	7,209,692	2	2	2	2	2	2	2	2
RC026	Chr4	9,488,466	9,565,569	9	15	12	5	14	7	10	7
RC027	Chr4	10,439,984	10,446,300	3	3	2	2	3	4	2	2
RC028	Chr4	10,625,562	10,657,282	4	4	4	6	6	4	4	4
RC029	Chr4	10,795,869	10,800,792	3	3	3	0	3	3	3	3
RC030	Chr4	12,236,593	12,272,940	3	3	3	3	3	3	3	3
RC031	Chr4	13,620,977	13,637,119	1	1	2	1	1	1	1	1
RC032	Chr4	17,098,612	17,108,825	2	3	2	2	2	2	2	2
RC033	Chr5	5,908,834	5,951,750	3	3	3	1	3	3	4	3
RC034	Chr5	6,074,069	6,089,041	3	3	3	2	2	2	2	2
RC035	Chr5	15,320,375	15,331,528	3	3	3	3	3	3	3	3
RC036	Chr5	16,034,441	16,047,572	3	3	3	3	3	3	3	3
RC037	Chr5	16,612,659	16,620,824	2	1	2	1	2	2	2	2
RC038	Chr5	16,688,626	16,699,028	1	1	2	1	2	2	2	2
RC039	Chr5	17,560,179	17,569,148	2	2	2	1	1	1	2	1
RC040	Chr5	18,114,461	18,205,000	8	8	10	10	8	8	8	8
RC041	Chr5	18,283,663	18,332,920	11	8	7	5	9	11	9	11
RC042	Chr5	18,412,059	18,432,430	4	5	2	3	3	4	5	2
RC043	Chr5	18,758,699	18,769,090	2	2	2	2	2	2	2	2
RC044	Chr5	18,835,605	18,872,494	4	2	5	3	3	4	4	3
RC045	Chr5	19,185,698	19,195,559	2	2	3	2	3	3	2	2
RC046	Chr5	19,773,117	19,779,815	0	2	2	0	2	0	2	0
RC047	Chr5	26,712,944	26,721,377	3	3	3	3	3	3	3	3

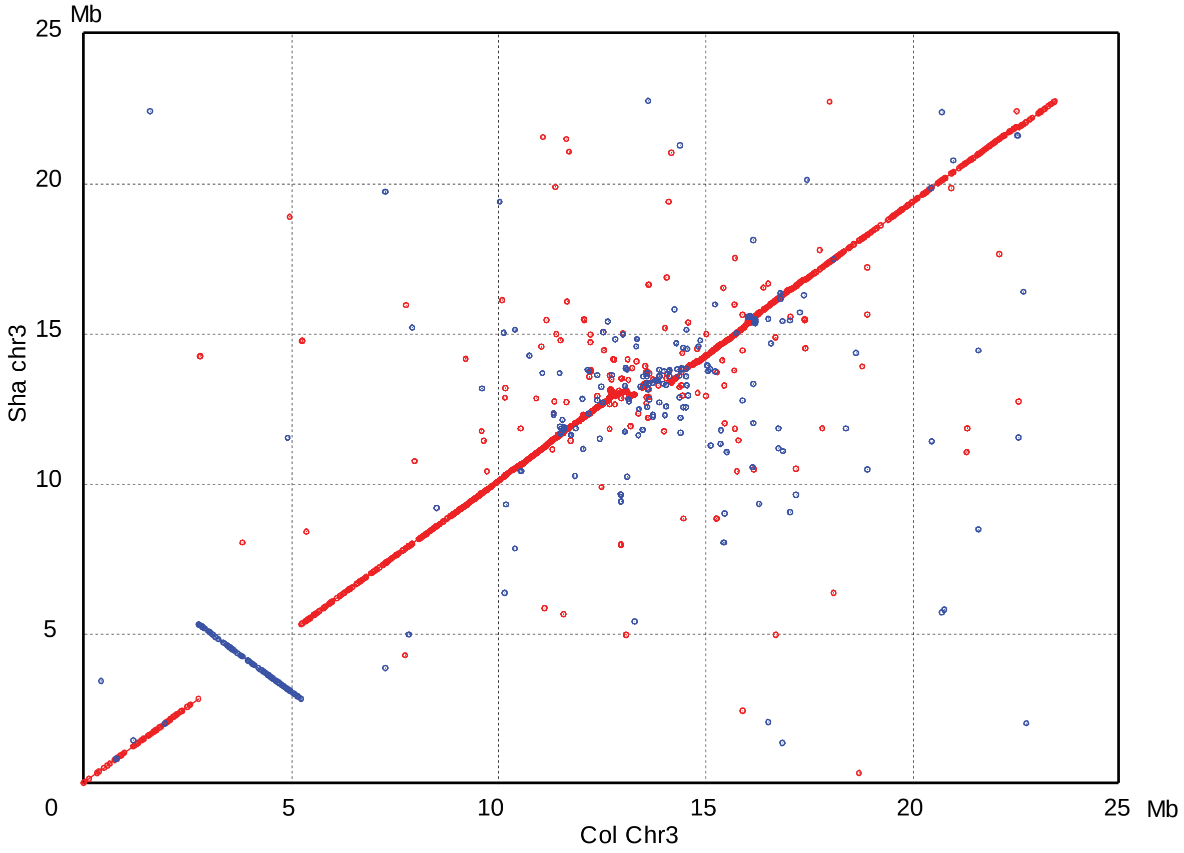

Supplementary Figure 1. A 2.48Mb inversion between the Col-0 reference and the Sha assembly.

The alignment was performed with nucmer with the parameter setting "-mum -c 40 -b 90 -l 100 -b 200". The plot was drawn using MUMmerplot [29]. Red: forward alignments, Blue: reverse alignments. Source Data are provided as a Source Data file.

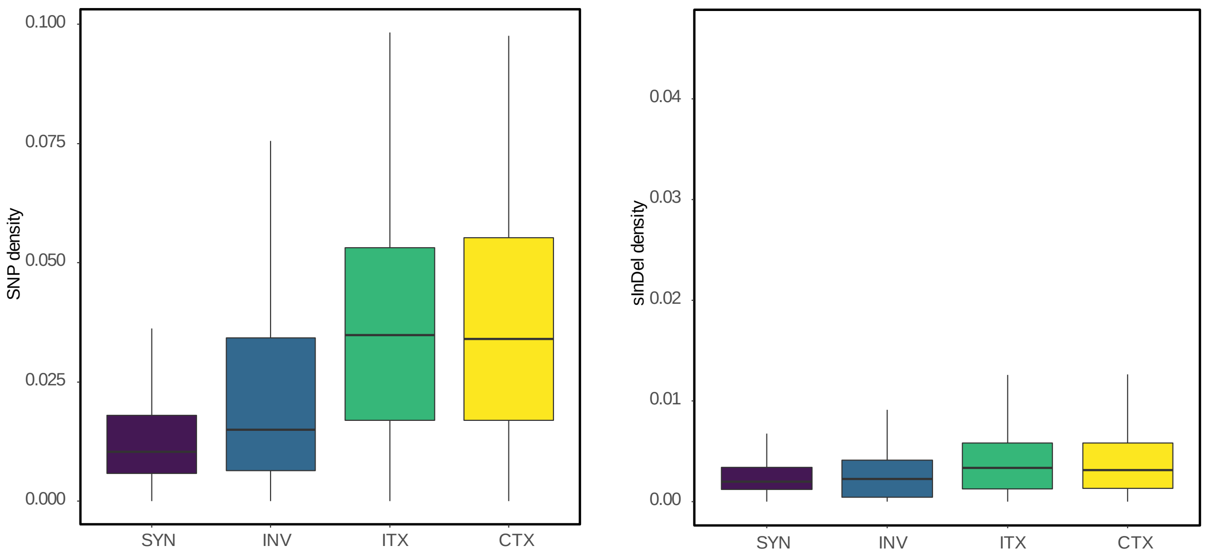

Supplementary Figure 2. Sequence variation density per bp in syntenic and rearranged regions.

Variations include SNPs and small InDels. Syntenic regions (SYN) number: n = 18,785; Inversion (INV) number: n = 3,350; intra-chromosome translocation (ITX) number: n = 3,150; inter-chromosome translocation (CTX) number: n = 3,363. In box plots, centre line: median, bounds of box: 25th and 75th percentiles, whiskers: 1.5 * IQR (IQR: the interquartile range between the 25th and the 75th percentile). Source Data are provided as a Source Data file.

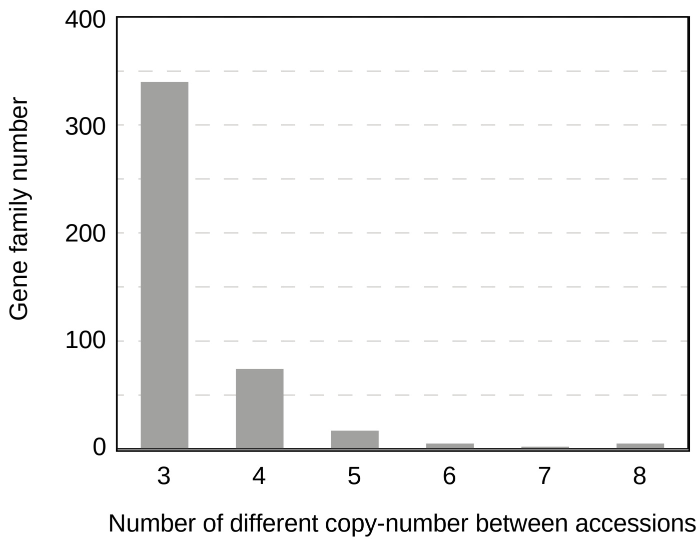

Supplementary Figure 3. Gene families with multiple different copy numbers across the eight accessions.

Source Data are provided as a Source Data file.

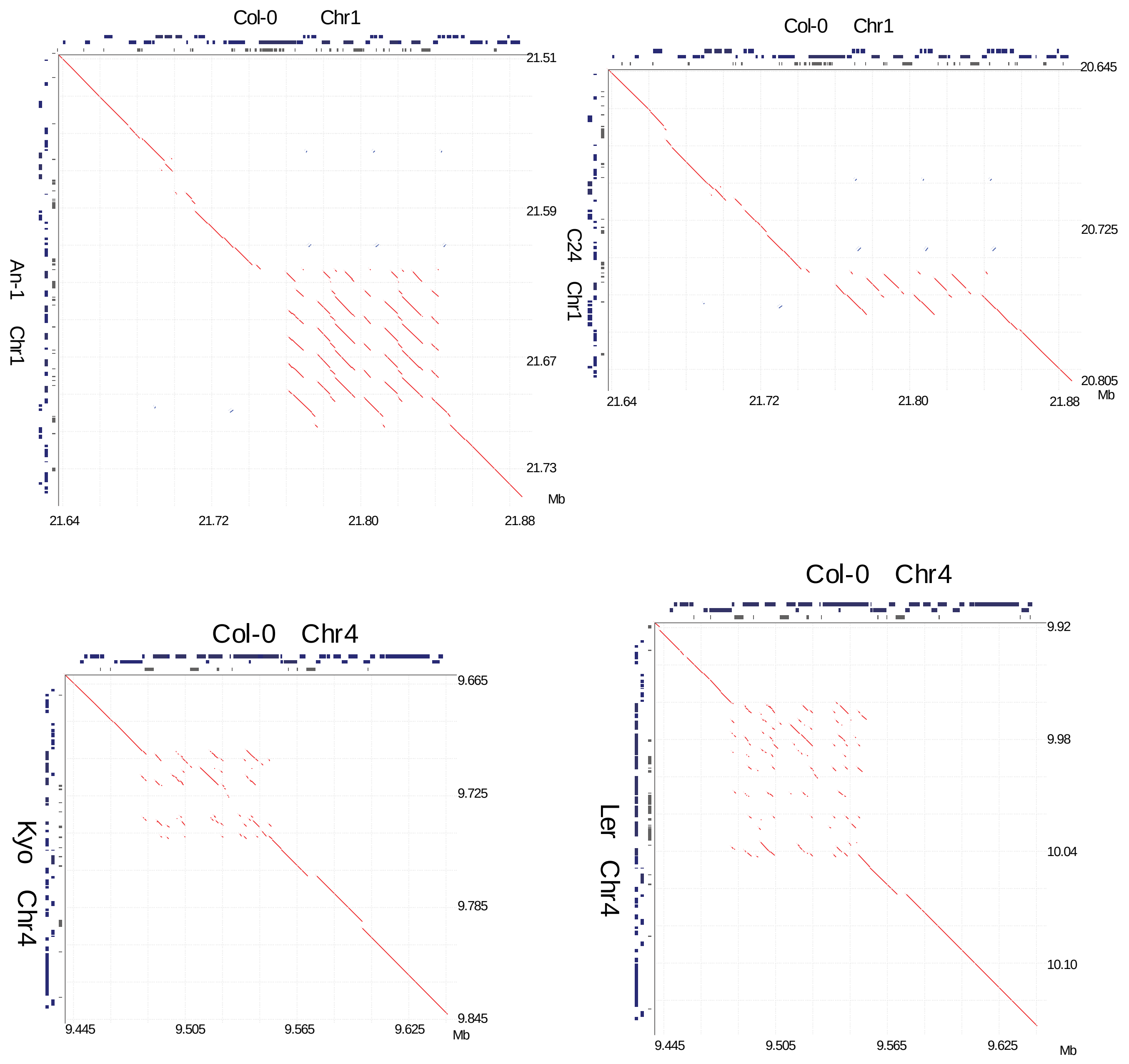

Supplementary Figure 4. Examples of sequence alignment dot plots of four hotspots of rearrangements.

The three rows on top and the three columns on the right show the location of genes on the forward strand (top), on the reverse strand (middle) and the repeat regions (bottom). Red line: forward alignment, blue line: reverse alignment. Source Data are provided as a Source Data file.

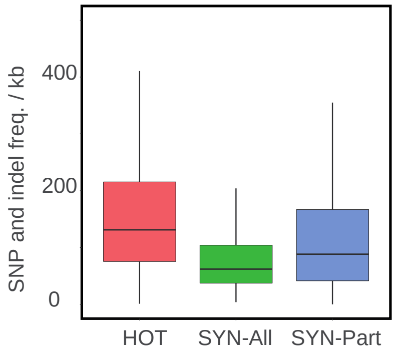

Supplementary Figure 5. Small variation frequency in three genomic regions.
The three genomic regions include HOT, SYN-All and SYN-Part. The small sequence variation was identified based on the whole genome alignments between each of seven accessions and Col-0 reference genome. HOT: regions with synteny diversity values of larger than 0.5, n = 576; SYN-All: regions which are syntenic in all pairwise comparisons (synteny diversity = 0), n = 10,331; SYN-Part: partially syntenic regions, including all regions remaining after excluding SYN-All and HOT regions, n = 10,501. In box plots, centre line: median, bounds of box: 25th and 75th percentiles, whiskers: 1.5 * IQR (IQR: the interquartile range between the 25th and the 75th percentile). Source Data are provided as a Source Data file.

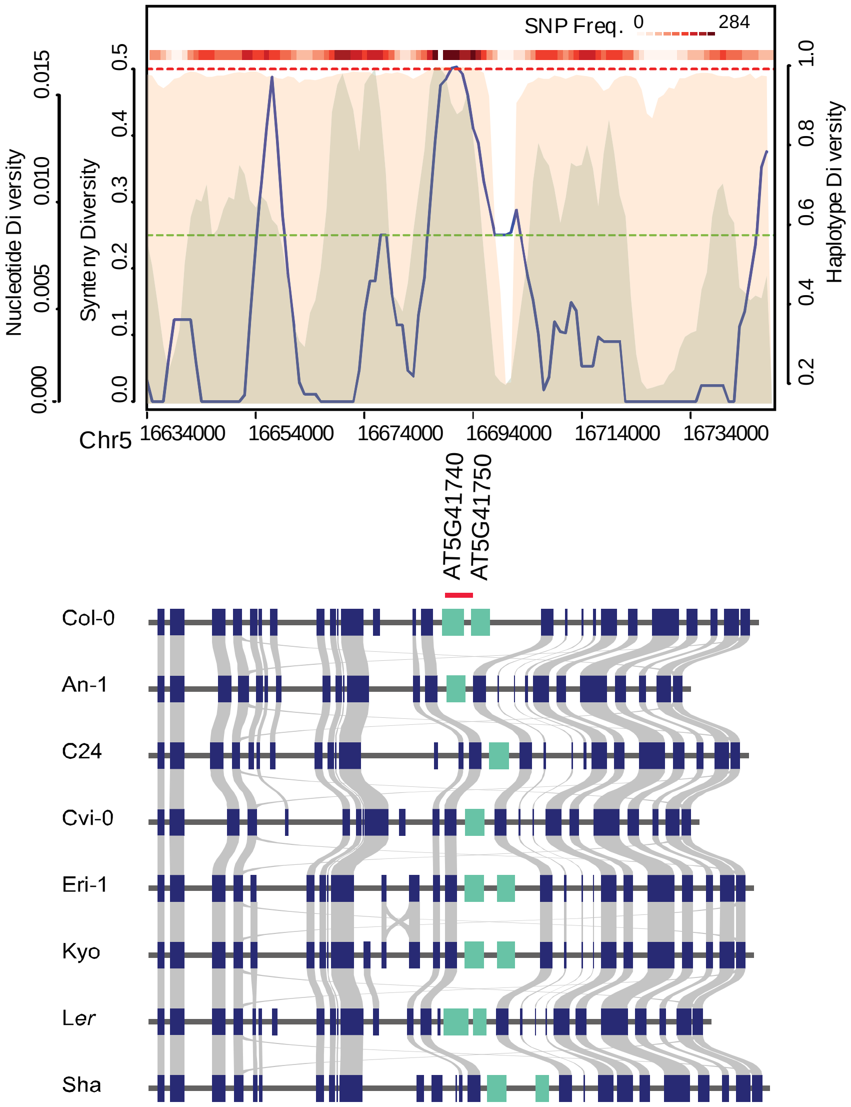

Supplementary Figure 6. Gene arrangements and variation analyses in the *Dangerous Mix 1* locus.

Variation analyses include synteny, nucleotide, and haplotype diversity analyses. *Dangerous Mix 1* (*DM1*) contains two tandem duplicated genes AT5G41740 and AT5G41750 [30]. One HOT region (shown by a red line above Col-0 genes) overlaps with *DM1*. Source Data are provided as a Source Data file.

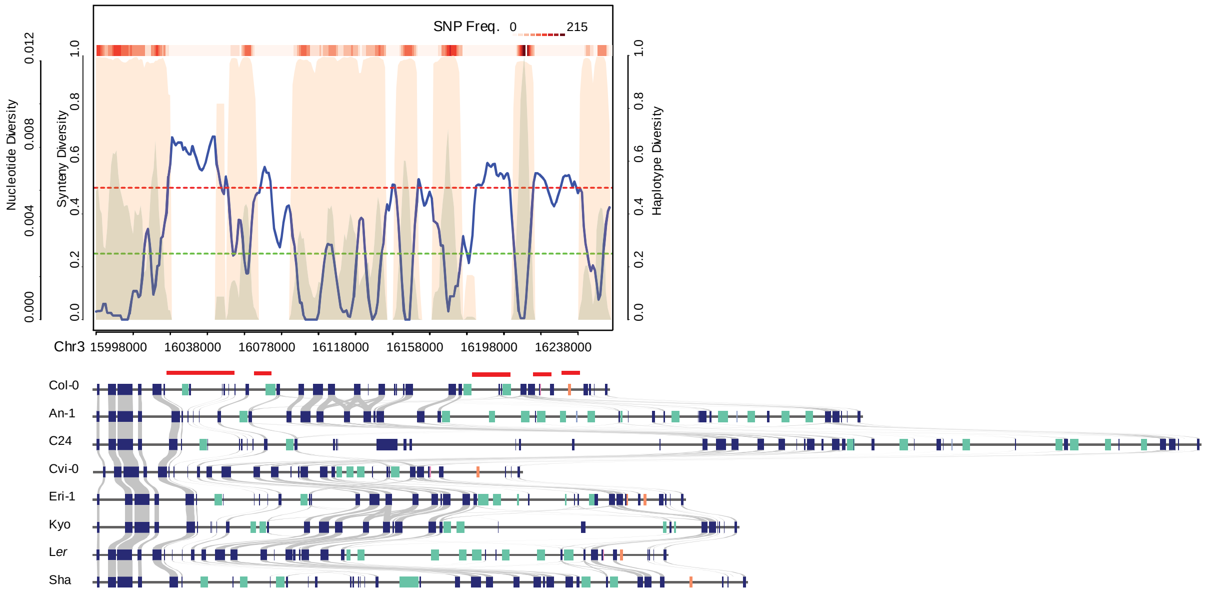

Supplementary Figure 7. Gene arrangements and variation analyses in the *Dangerous Mix 2* locus.

Variation analyses include synteny, nucleotide, and haplotype diversity analyses. *Dangerous Mix 2* (*DM2*) was mapped to chromosome 3:16.15 - 16.30 Mb [31]. Several HOT regions (shown by red lines above Col-0 genes) are in or around this locus. Source Data are provided as a Source Data file.

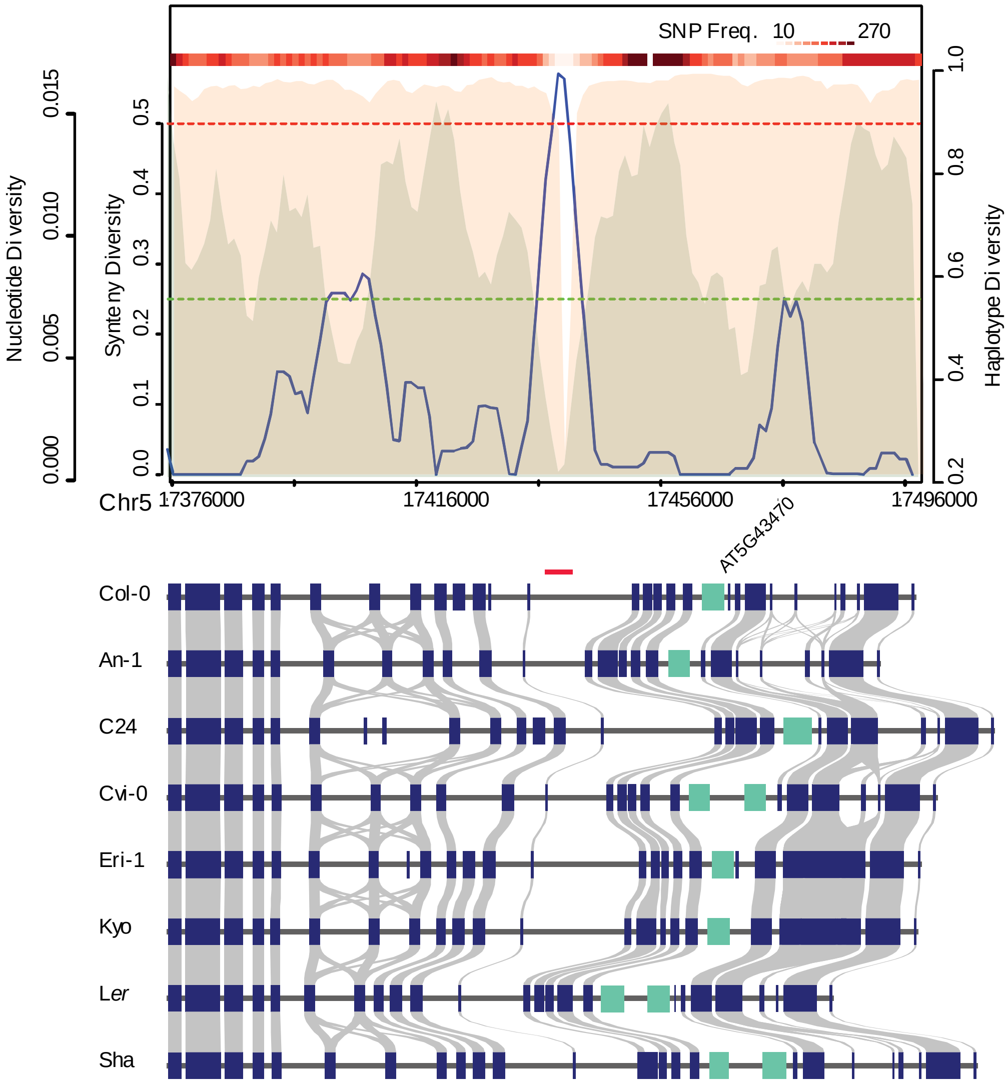

Supplementary Figure 8. Gene arrangements and variation analyses in the *Dangerous Mix 4* locus.

Variation analyses include synteny, nucleotide, and haplotype diversity analyses. *Dangerous Mix 4* (*DM4*) locus was mapped to chromosome 5:17.16 - 17.50 Mb [31]. One HOT region (shown by a red line above Col-0 genes) is in this locus. Source Data are provided as a Source Data file.

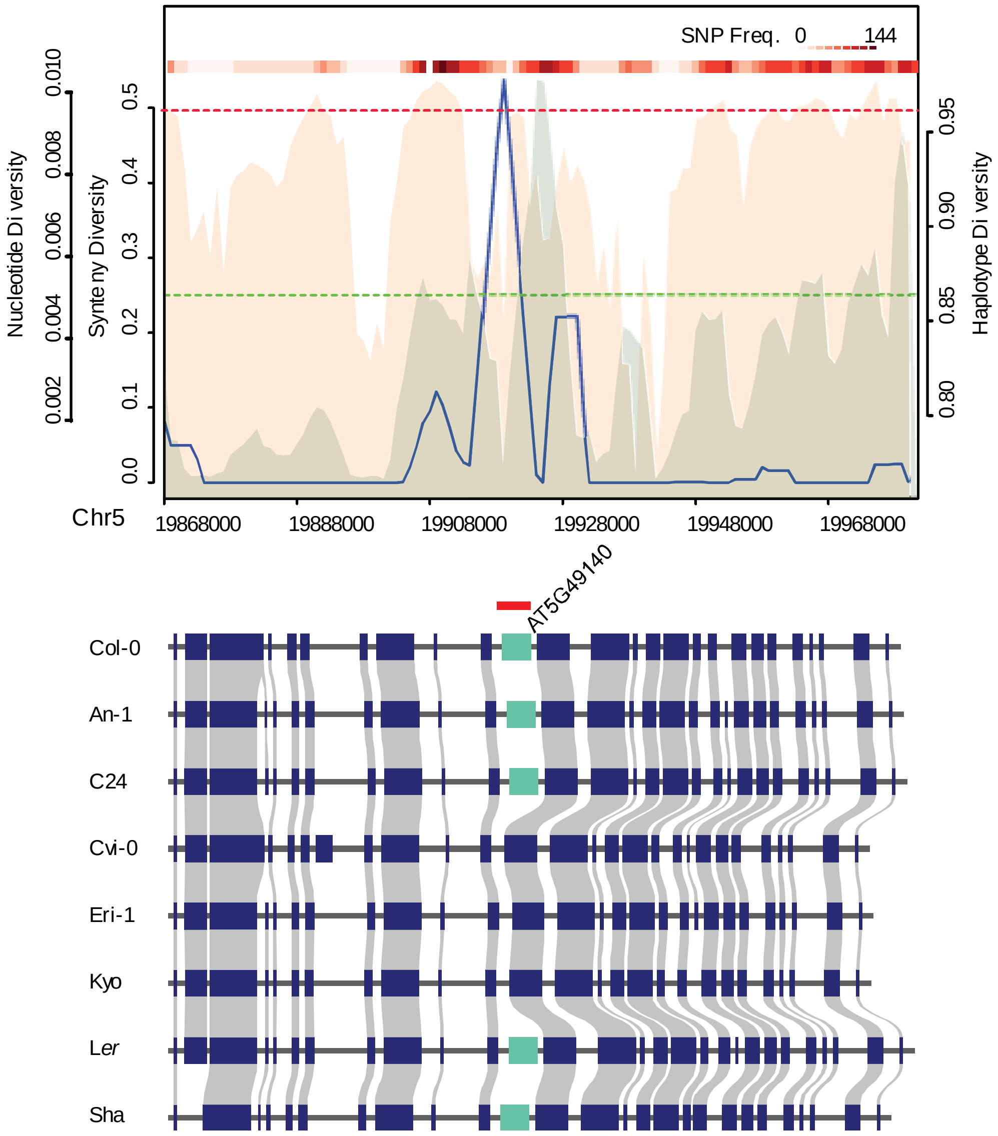

Supplementary Figure 9. Gene arrangements and variation analyses in the *Dangerous Mix 5* locus.

Variation analyses include synteny, nucleotide, and haplotype diversity analyses. *Dangerous Mix 5* (*DM5*) locus was mapped to chromosome 5:19.89 - 20.48 Mb [31]. One HOT region (shown by red line above Col-0 genes) is in this locus. Source Data are provided as a Source Data file.

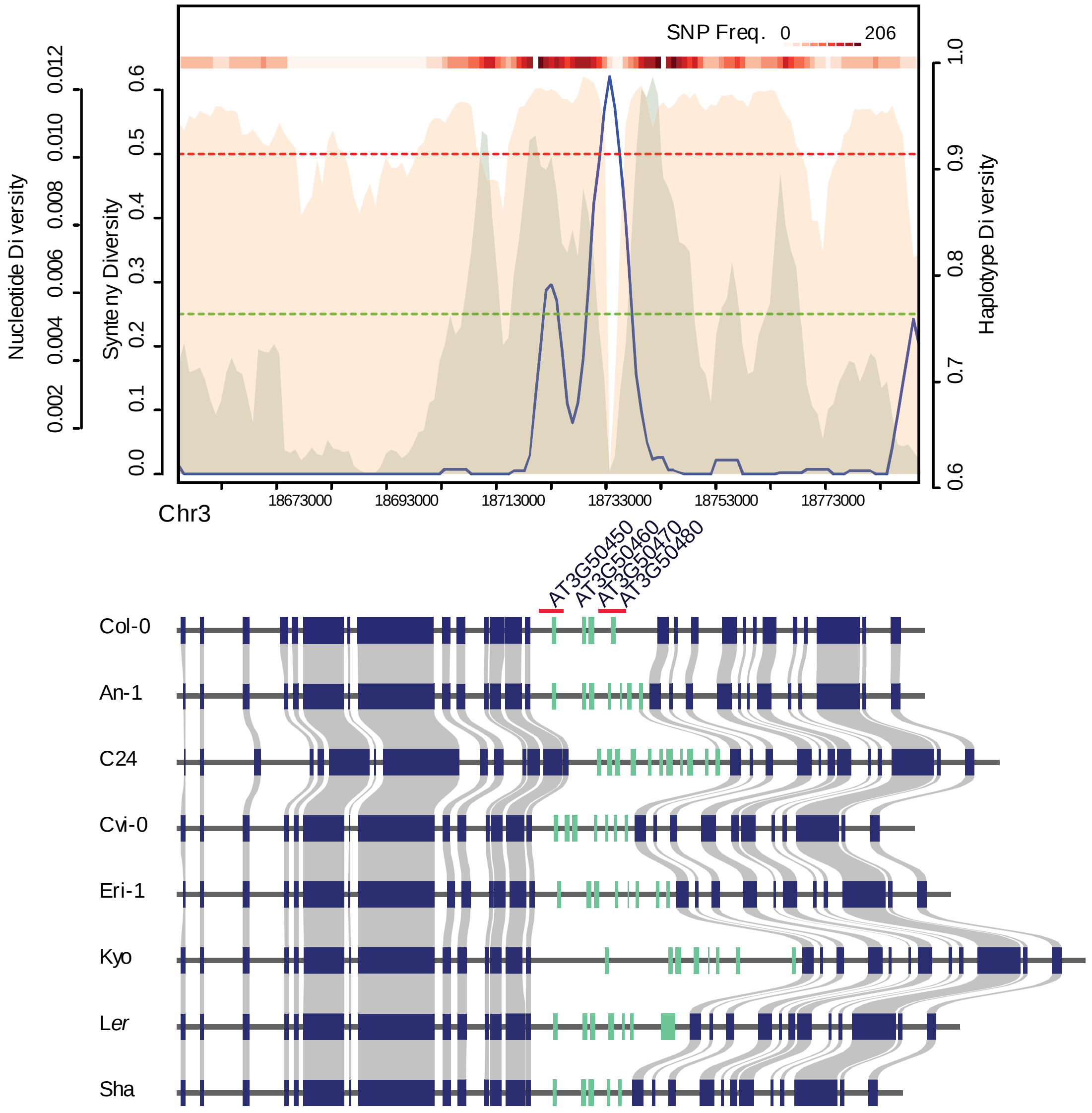

Supplementary Figure 10. Gene arrangements and variation analyses in the *Dangerous Mix 7* locus.

Variation analyses include synteny, nucleotide, and haplotype diversity analyses. *Dangerous Mix 7* (*DM7*) locus was mapped to two intervals on chromosome 3:18.53 – 18.99 Mb and 18.58-18.93 Mb [31]. Two HOT regions (shown by red lines above Col-0 genes) overlapped with this interval and the R gene cluster *RPW8.1/RPW8.2*. Source Data are provided as a Source Data file.

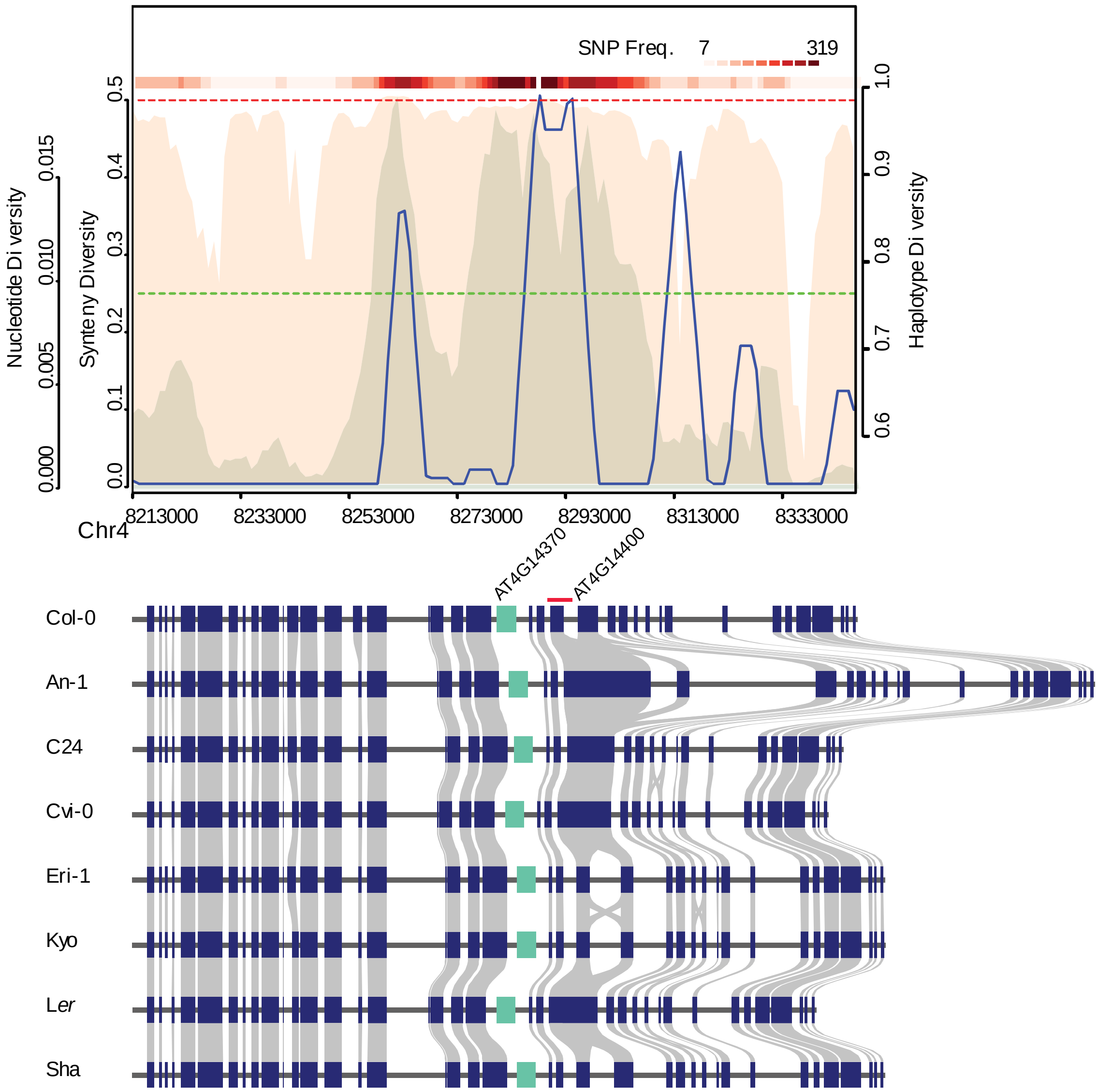

Supplementary Figure 11. Gene arrangements and variation analyses in the *Dangerous Mix 9* locus.

Variation analyses include synteny, nucleotide, and haplotype diversity analyses. A small HOT region (shown by a red line above Col-0 genes) overlaps with the *Dangerous Mix 9* (*DM9*) *ACD6* allele (AT4G14400) [32]. Source Data are provided as a Source Data file.

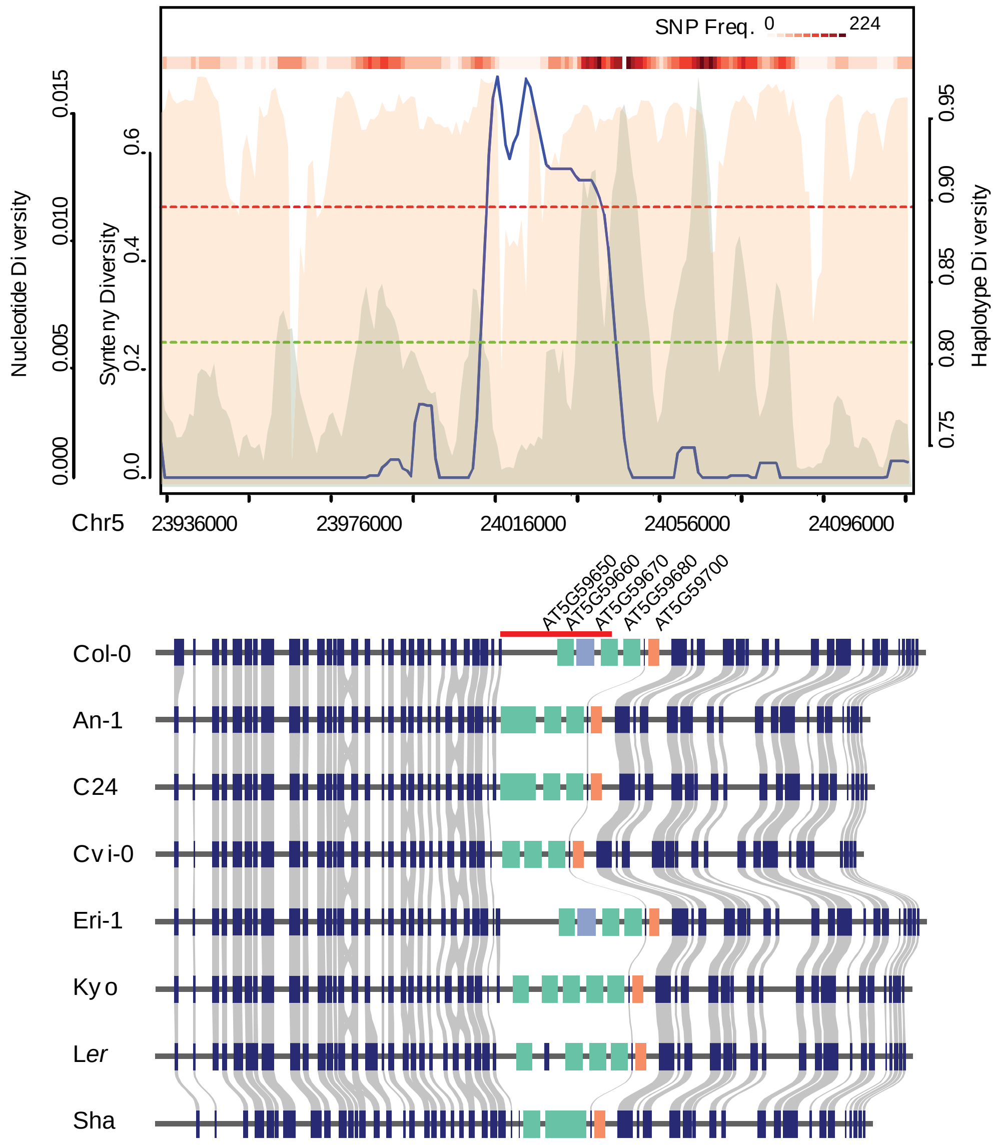

Supplementary Figure 12. Gene arrangements and variation analyses in the single-locus genetic incompatibility locus of the OAK gene.

Variation analyses include synteny, nucleotide, and haplotype diversity analyses. A large HOT region (shown by a red line above Col-0 genes) overlaps with the *OAK* gene including four receptor-like kinases (AT5G59650 to AT5G59680) [33]. Source Data are provided as a Source Data file.

Supplementary References

1. Simon, M. *et al.* Quantitative trait loci mapping in five new large recombinant inbred line populations of *Arabidopsis thaliana* genotyped with consensus single-nucleotide polymorphism markers. *Genetics* **178**, 2253-2264 (2008).
2. Singer, T. *et al.* A high-resolution map of *Arabidopsis* recombinant inbred lines by whole-genome exon array hybridization. *PLoS Genet.* **2**, e144 (2006).
3. Giraut, L. *et al.* Genome-wide crossover distribution in *Arabidopsis thaliana* meiosis reveals sex-specific patterns along chromosomes. *PLoS Genet.* **7**, e1002354 (2011).
4. Clauw, P. *et al.* Leaf responses to mild drought stress in natural variants of *Arabidopsis*. *Plant Physiol.* **167**, 800-816 (2015).
5. Clauw, P. *et al.* Leaf growth response to mild drought: natural variation in *Arabidopsis* sheds light on trait architecture. *Plant Cell* **28**, 2417-2434 (2016).
6. Van Veen, H. *et al.* Transcriptomes of eight *Arabidopsis thaliana* accessions reveal core conserved, genotype- and organ-specific responses to flooding stress. *Plant Physiology* **172**, 668-689 (2016).
7. Cui, P., Zhang, S., Ding, F., Ali, S. & Xiong, L. Dynamic regulation of genome-wide pre-mRNA splicing and stress tolerance by the Sm-like protein LSm5 in *Arabidopsis*. *Genome Biol.* **15**, R1 (2014).
8. Miller, M., Song, Q., Shi, X., Juenger, T. E. & Chen, Z. J. Natural variation in timing of stress-responsive gene expression predicts heterosis in intraspecific hybrids of *Arabidopsis*. *Nat. Commun.* **6**, 7453 (2015).
9. Ding, F. *et al.* Genome-wide analysis of alternative splicing of pre-mRNA under salt stress in *Arabidopsis*. *BMC Genomics* **15**, 431 (2014).
10. Zhao, Y. *et al.* *REPRESSOR OF SILENCING5* encodes a member of the small heat shock protein family and is required for DNA demethylation in *Arabidopsis* . *Plant Cell* **26**, 2660-2675 (2014).
11. Perez-Santángelo, S. *et al.* Role for LSM genes in the regulation of circadian rhythms . *Proc. Natl. Acad. Sci.* **111**, 15166-15171 (2014).
12. Xu, E. *et al.* Quantitative trait loci mapping and transcriptome analysis reveal candidate genes regulating the response to ozone in *Arabidopsis thaliana*. *Plant, Cell Environ.* **38**, 1418-1433 (2015).
13. Cui, P. *et al.* The RNA polymerase II C-terminal domain phosphatase-like protein FIERY2/CPL1 interacts with eIF4AIII and is essential for nonsense-mediated mRNA decay in *Arabidopsis*. *Plant Cell* **28**, 770-85 (2016).
14. Du, J. L. *et al.* The splicing factor PRP31 is Involved in transcriptional gene silencing and stress response in *Arabidopsis*. *Mol. Plant* **8**, 1053-1068 (2015).

15. Groszmann, M. *et al.* Hormone-regulated defense and stress response networks contribute to heterosis in *Arabidopsis* F1 hybrids . *Proc. Natl. Acad. Sci.* **112**, E6397-E6406 (2015).
16. Wang, L. *et al.* Hybrid mimics and hybrid vigor in *Arabidopsis* . *Proc. Natl. Acad. Sci.* **112**, E4959-E4967 (2015).
17. Piofczyk, T., Jeena, G. & Pecinka, A. *Arabidopsis thaliana* natural variation reveals connections between UV radiation stress and plant pathogen-like defense responses. *Plant Physiol. Biochem.* **93**, 34-43 (2015).
18. Zhang, Q. *et al.* The chromatin remodeler DDM1 promotes hybrid vigor by regulating salicylic acid metabolism. *Cell Discov.* **2**, 16027 (2016).
19. Kawakatsu, T. *et al.* Epigenomic diversity in a global collection of *Arabidopsis thaliana* accessions. *Cell* **166**, 492–505 (2016).
20. Wang, L. *et al.* PIF4-controlled auxin pathway contributes to hybrid vigor in *Arabidopsis thaliana*. *Proc. Natl. Acad. Sci.* **114**, E3555-E3562 (2017).
21. Leydon, A. R. *et al.* The molecular dialog between flowering plant reproductive partners defined by SNP-informed RNA-sequencing. *Plant Cell* **29**, 984-1006 (2017).
22. Picard, C. L. & Gehring, M. Proximal methylation features associated with nonrandom changes in gene body methylation. *Genome Biol.* **18**, 73 (2017).
23. Li, Q., Li, Y., Moose, S. P. & Hudson, M. E. Transposable elements, mRNA expression level and strand-specificity of small RNAs are associated with non-additive inheritance of gene expression in hybrid plants. *BMC Plant Biol.* **15**, 168 (2015).
24. Li, D. *et al.* FAR-RED ELONGATED HYPOCOTYL3 activates SEPALLATA2 but inhibits CLAVATA3 to regulate meristem determinacy and maintenance in *Arabidopsis*. *Proc. Natl. Acad. Sci.* **113**, 9375-9380 (2016).
25. Wuest, S. E., Philipp, M. A., Guthörl, D., Schmid, B. & Grossniklaus, U. Seed production affects maternal growth and senescence in *Arabidopsis*. *Plant Physiol.* **171**, 392-404 (2016).
26. Huang, Z. *et al.* APETALA2 antagonizes the transcriptional activity of AGAMOUS in regulating floral stem cells in *Arabidopsis thaliana*. *New Phytol.* **215**, 1197-1209 (2017).
27. Ezer, D. *et al.* The G-box transcriptional regulatory code in *Arabidopsis*. *Plant Physiol.* **175**, 628-640 (2017).
28. Lei, L., Steffen, J. G., Osborne, E. J. & Toomajian, C. Plant organ evolution revealed by phylotranscriptomics in *Arabidopsis thaliana*. *Sci. Rep.* **7**, 7567 (2017).
29. Marçais, G. *et al.* MUMmer4: a fast and versatile genome alignment system. *PLoS Comput. Biol.* **14**, e1005944 (2018).

30. Bomblies, K. *et al.* Autoimmune response as a mechanism for a Dobzhansky-Muller-type incompatibility syndrome in plants. *PLoS Biol.* **5**, 1962–1972 (2007).
31. Chae, E. *et al.* Species-wide genetic incompatibility analysis identifies immune genes as hot spots of deleterious epistasis. *Cell* **159**, 1341–1351 (2014).
32. Todesco, M. *et al.* Activation of the *Arabidopsis thaliana* immune system by combinations of common *ACD6* alleles. *PLoS Genet.* **10**, e1004459 (2014).
33. Smith, L. M., Bomblies, K. & Weigel, D. Complex evolutionary events at a tandem cluster of *Arabidopsis thaliana* genes resulting in a single-locus genetic incompatibility. *PLoS Genet.* **7**, e1002164 (2011).
