# Supplementary material for: Chromosome-level assemblies of multiple Arabidopsis genomes reveal hotspots of rearrangements with altered evolutionary dynamics
Source: Nat Commun. 2020 Feb 20;11:989. doi: 10.1038/s41467-020-14779-y (PMC7033125; doi:10.1038/s41467-020-14779-y)
Supplement: Supplementary file 3 — Reporting Summary [file 41467_2020_14779_MOESM3_ESM.pdf]

nature research

Corresponding author(s): Prof Dr Korbinian Schneeberger

Last updated by author(s): Jan 7, 2020

Reporting Summary

Nature Research wishes to improve the reproducibility of the work that we publish. This form provides structure for consistency and transparency in reporting. For further information on Nature Research policies, see Authors & Referees and the Editorial Policy Checklist .

Statistics

For all statistical analyses, confirm that the following items are present in the figure legend, table legend, main text, or Methods section.

n/a	Confirmed	
☐	☒	The exact sample size (n) for each experimental group/condition, given as a discrete number and unit of measurement
☐	☒	A statement on whether measurements were taken from distinct samples or whether the same sample was measured repeatedly
☐	☒	The statistical test(s) used AND whether they are one- or two-sided<br>*Only common tests should be described solely by name; describe more complex techniques in the Methods section.*
☒	☐	A description of all covariates tested
☐	☒	A description of any assumptions or corrections, such as tests of normality and adjustment for multiple comparisons
☐	☒	A full description of the statistical parameters including central tendency (e.g. means) or other basic estimates (e.g. regression coefficient) AND variation (e.g. standard deviation) or associated estimates of uncertainty (e.g. confidence intervals)
☐	☒	For null hypothesis testing, the test statistic (e.g. F, t, r) with confidence intervals, effect sizes, degrees of freedom and P value noted<br>*Give P values as exact values whenever suitable.*
☒	☐	For Bayesian analysis, information on the choice of priors and Markov chain Monte Carlo settings
☒	☐	For hierarchical and complex designs, identification of the appropriate level for tests and full reporting of outcomes
☒	☐	Estimates of effect sizes (e.g. Cohen's d, Pearson's r), indicating how they were calculated

Our web collection on statistics for biologists contains articles on many of the points above.

Software and code

Policy information about availability of computer code

Data collection	No software was used to collect data
Data analysis	SMRTLink5, version5; Falcon, version 0.3.0; Canu, version 1.5; MECAT, version 1<br><br>BWA, version 0.7.12; SAMTools, version 1.9; MUMmer4, version 4; Blast version 2.2.29<br><br>Augustus version 3.2.3; GlimmerHMM version 3.0.3; SNAP version 2006-07-28<br><br>HISAT2 version 2.1.0; StringTie version v1.3.4d; Evidence Modeler version r2012-06-25; Infernal version 1.1; RGAugury version 1<br><br>snpEff version 4.3p; OrthoFinder version 2.2.6; PopGenome version 2.7.1<br><br>DAVID version 6.8; Scipio version 1.4; SyRI version 1.1; RepeatMasker version 4.0.7; exonerate version 2.2.0<br><br>R version 3.5.2; PERL version 5.22.2; Python version 2.7.15<br><br>custom scripts: https://github.com/schneebergerlab/AMPRIL-genomes

For manuscripts utilizing custom algorithms or software that are central to the research but not yet described in published literature, software must be made available to editors/reviewers. We strongly encourage code deposition in a community repository (e.g. GitHub). See the Nature Research guidelines for submitting code & software for further information.

Data

Policy information about availability of data

All manuscripts must include a data availability statement. This statement should provide the following information, where applicable:

- Accession codes, unique identifiers, or web links for publicly available datasets
- A list of figures that have associated raw data
- A description of any restrictions on data availability

Raw sequencing data, assemblies and annotations can be accessed in the European Nucleotide Archive under the project accession number PRJEB31147. Assemblies, annotation, variation and orthologs can be found on the 1001 Arabidopsis thaliana Genomes webpage https://1001genomes.org/data/MPIPZ/MPIPZJiao2020/releases/current/. RNA-seq data from the five accessions (An-1, C24, Cvi-0, Ler, and Sha) are downloaded from the NCBI SRA database (the NCBI and ENA accession codes are included in Supplementary Data 2). The SNP markers resulted from 1001 Genomes Project can be downloaded from the webpage https://1001genomes.org/data/GMI-MPI/releases/v3.1/. Data supporting the findings of this work are available within the paper and its Supplementary Information files. A reporting summary for this Article is available as a Supplementary Information file. The datasets generated and analyzed during the current study are available from the corresponding author upon request. The source data underlying Figures 1, 2, 3, 4 and 5, as well as Supplementary Figures 1-12 are provided as a Source Data file "Source Data.zip".

Field-specific reporting

Please select the one below that is the best fit for your research. If you are not sure, read the appropriate sections before making your selection.

- [x] Life sciences
- [ ] Behavioural & social sciences
- [ ] Ecological, evolutionary & environmental sciences

For a reference copy of the document with all sections, see nature.com/documents/nr-reporting-summary-flat.pdf

Life sciences study design

All studies must disclose on these points even when the disclosure is negative.

Sample size	Seven Arabidopsis thaliana accessions from geographically diverse populations including An-1 (Antwerpen, Belgium), C24 (Coimbra, Portugal), Cvi-0 (Cape Verde Islands), Eri-1 (Eringsboda, Sweden), Kyo (Kyoto, Japan), Ler (Gorzów Wielkopolski, Poland) and Sha (Shahdara, Tadjikistan). These accessions together with Col-0 accession are the eight founders of an Arabidopsis thaliana multiparent RIL (recombination inbreeding line) population. These accessions are distributed across the global range of this predominantly inbred plant.
Data exclusions	PacBio reads were filtered for short (<50bp) or low quality (QV<80) reads using SMRTLink5 package
Replication	The same pipeline for genome assembly and gene annotation was done for each of seven genomes.
Randomization	Not relevant. Because data generated from the samples were used without allocation.
Blinding	Not relevant. Because no group allocation was done during data collection and analysis.

Reporting for specific materials, systems and methods

We require information from authors about some types of materials, experimental systems and methods used in many studies. Here, indicate whether each material, system or method listed is relevant to your study. If you are not sure if a list item applies to your research, read the appropriate section before selecting a response.

Materials & experimental systems

n/a	Involved in the study	
[x]	[ ]	Antibodies
[x]	[ ]	Eukaryotic cell lines
[x]	[ ]	Palaeontology
[x]	[ ]	Animals and other organisms
[x]	[ ]	Human research participants
[x]	[ ]	Clinical data

Methods

n/a	Involved in the study	
[x]	[ ]	ChIP-seq
[x]	[ ]	Flow cytometry
[x]	[ ]	MRI-based neuroimaging
