# Supplementary material for: Chromosome-level assemblies of multiple Arabidopsis genomes reveal hotspots of rearrangements with altered evolutionary dynamics
Source: Nat Commun. 2020 Feb 20;11:989. doi: 10.1038/s41467-020-14779-y (PMC7033125; doi:10.1038/s41467-020-14779-y)
Supplement: Supplementary file 4 — Description of Additional Supplementary Files [file 41467_2020_14779_MOESM4_ESM.pdf]

Description of Additional Supplementary Files

File Name: Supplementary Data 1
Description: Location of centromeric and telomeric tandem repeat arrays

File Name: Supplementary Data 2
Description: RNA-seq data used for gene annotations

File Name: Supplementary Data 3
Description: List of HOT regions
