# Supplementary material for: Chromosome-level assemblies of multiple Arabidopsis genomes reveal hotspots of rearrangements with altered evolutionary dynamics
Source: Nat Commun. 2020 Feb 20;11:989. doi: 10.1038/s41467-020-14779-y (PMC7033125; doi:10.1038/s41467-020-14779-y)
Supplement: Supplementary file 8 — Source Data [file 41467_2020_14779_MOESM8_ESM.zip › Source_Data.SupplementaryFigure2.zip!/Source_Data.SupplementaryFigure2/AMPRIL.wga.SYN.GR.local.var.density.boxplot.snp.pdf]

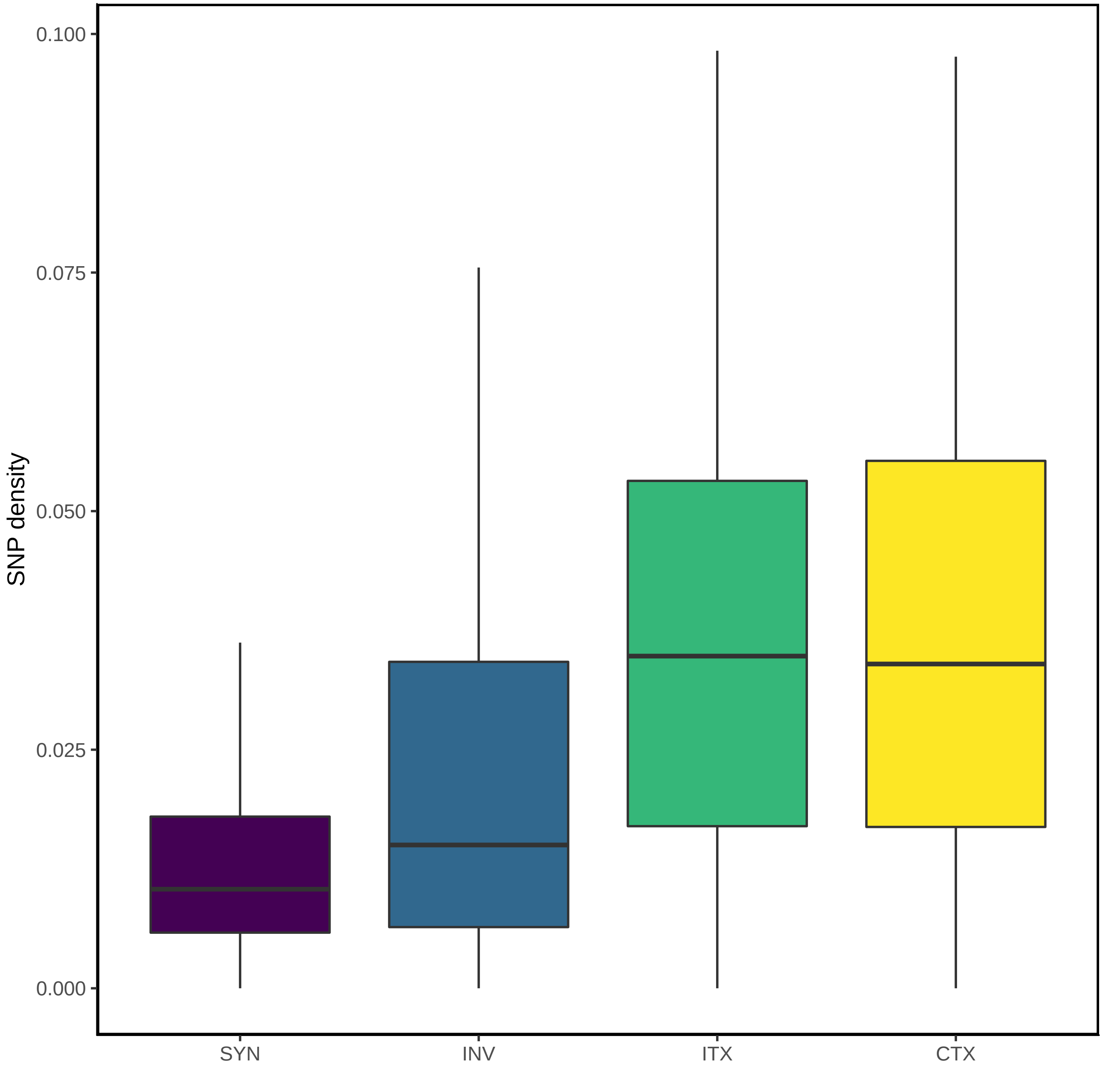
SNP density
0.100
0.075
0.050
0.025
0.000
SYN
INV
ITX
CTX
