# Supplementary material for: Chromosome-level assemblies of multiple Arabidopsis genomes reveal hotspots of rearrangements with altered evolutionary dynamics
Source: Nat Commun. 2020 Feb 20;11:989. doi: 10.1038/s41467-020-14779-y (PMC7033125; doi:10.1038/s41467-020-14779-y)
Supplement: Supplementary file 8 — Source Data [file 41467_2020_14779_MOESM8_ESM.zip › Source_Data.SupplementaryFigure1.zip!/Source_Data.SupplementaryFigure1/SupFig2.Sha.Chr3.pdf]

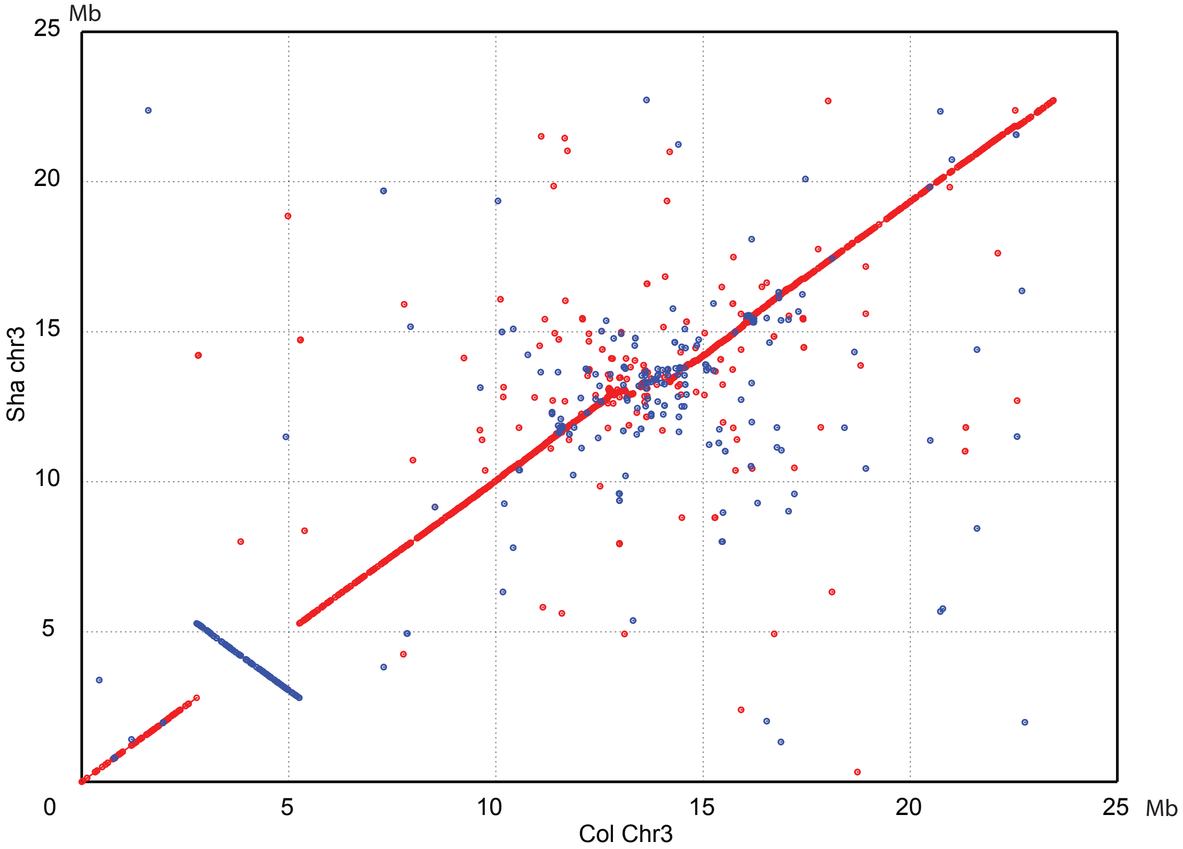

25
Mb
20
15
10
5
0
5
10
15
20
25
Mb
Sha chr3
Col Chr3
