# Supplementary material for: Chromosome-level assemblies of multiple Arabidopsis genomes reveal hotspots of rearrangements with altered evolutionary dynamics
Source: Nat Commun. 2020 Feb 20;11:989. doi: 10.1038/s41467-020-14779-y (PMC7033125; doi:10.1038/s41467-020-14779-y)
Supplement: Supplementary file 8 — Source Data [file 41467_2020_14779_MOESM8_ESM.zip › Source_Data.SupplementaryFigure5.zip!/Source_Data.SupplementaryFigure5/AMPRIL.HDR.vs.SYN-All.var.all.eff.chrArm.snp.indel.density.boxplot.pdf]

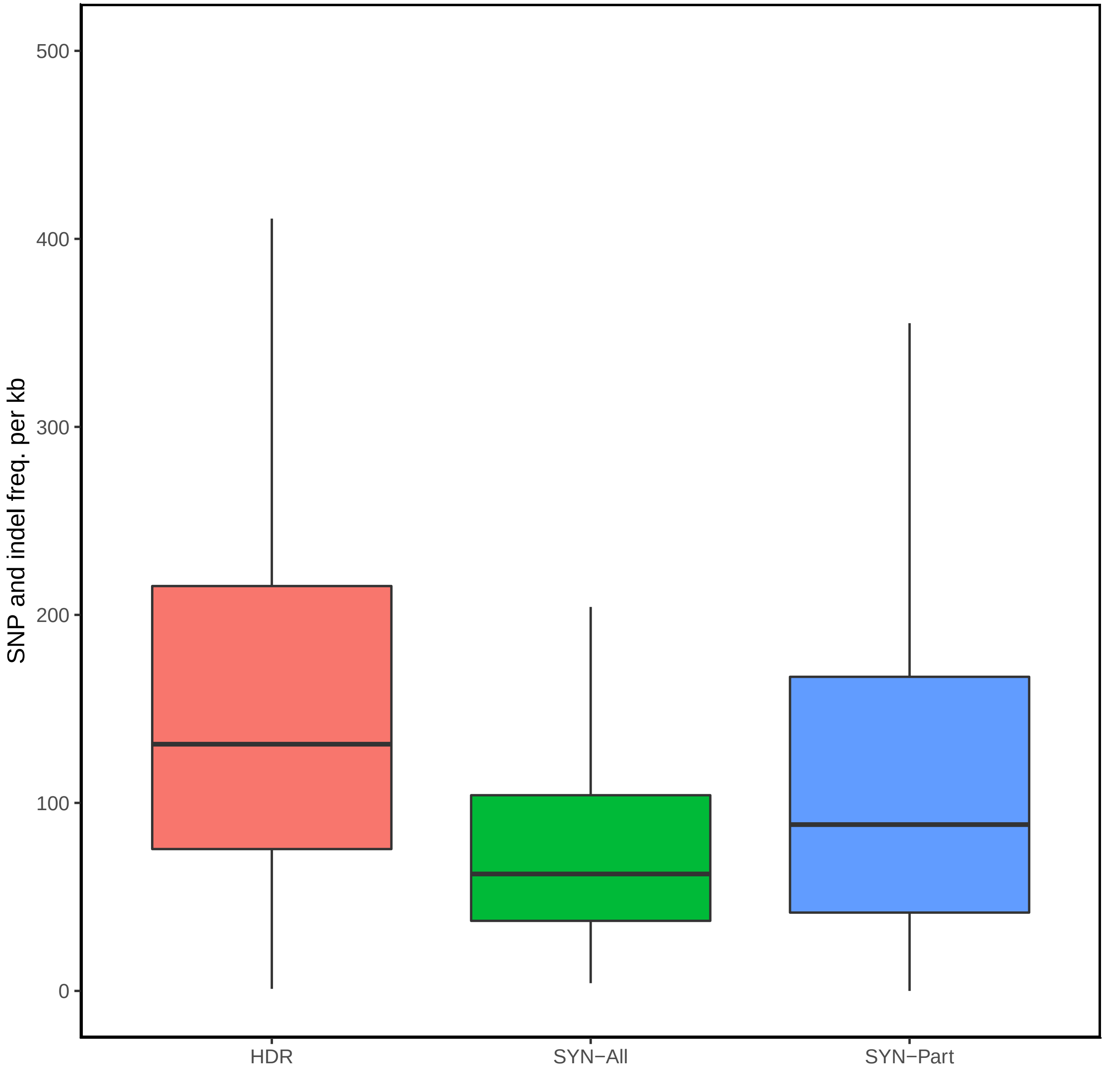
SNP and indel freq. per kb
500
400
300
200
100
0
HDR
SYN−All
SYN−Part
