# Supplementary material for: Chromosome-level assemblies of multiple Arabidopsis genomes reveal hotspots of rearrangements with altered evolutionary dynamics
Source: Nat Commun. 2020 Feb 20;11:989. doi: 10.1038/s41467-020-14779-y (PMC7033125; doi:10.1038/s41467-020-14779-y)
Supplement: Supplementary file 8 — Source Data [file 41467_2020_14779_MOESM8_ESM.zip › Source_Data.SupplementaryFigure6.zip!/Source_Data.SupplementaryFigure6/Chr5_16690001_16696000.win5kb.step1kb.MAF005.pdf]

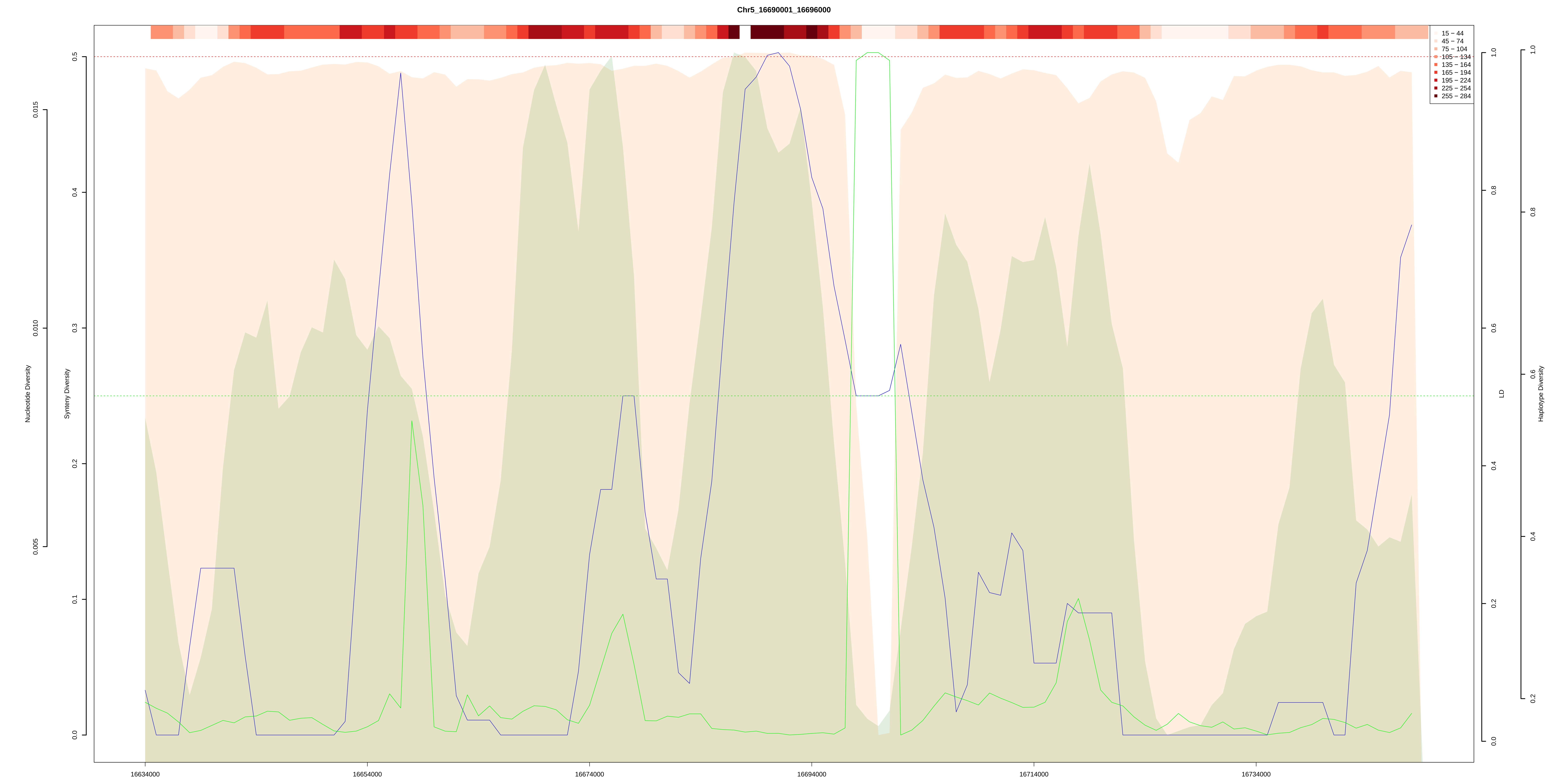

Chr5_16690001_16696000
15 – 44
45 – 74
75 – 104
105 – 134
135 – 164
165 – 194
195 – 224
225 – 254
255 – 284
Nucleotide Diversity
Synteny Diversity
LD
Haplotype Diversity
0.005
0.010
0.015
0.0
0.1
0.2
0.3
0.4
0.5
0.6
0.8
1.0
16634000
16654000
16674000
16694000
16714000
16734000
