# Supplementary material for: Chromosome-level assemblies of multiple Arabidopsis genomes reveal hotspots of rearrangements with altered evolutionary dynamics
Source: Nat Commun. 2020 Feb 20;11:989. doi: 10.1038/s41467-020-14779-y (PMC7033125; doi:10.1038/s41467-020-14779-y)
Supplement: Supplementary file 8 — Source Data [file 41467_2020_14779_MOESM8_ESM.zip › Source_Data.SupplementaryFigure7.zip!/Source_Data.SupplementaryFigure7/RPP1_DM2.win5kb.step1kb.popGenome.miss02.MAF005.pdf]

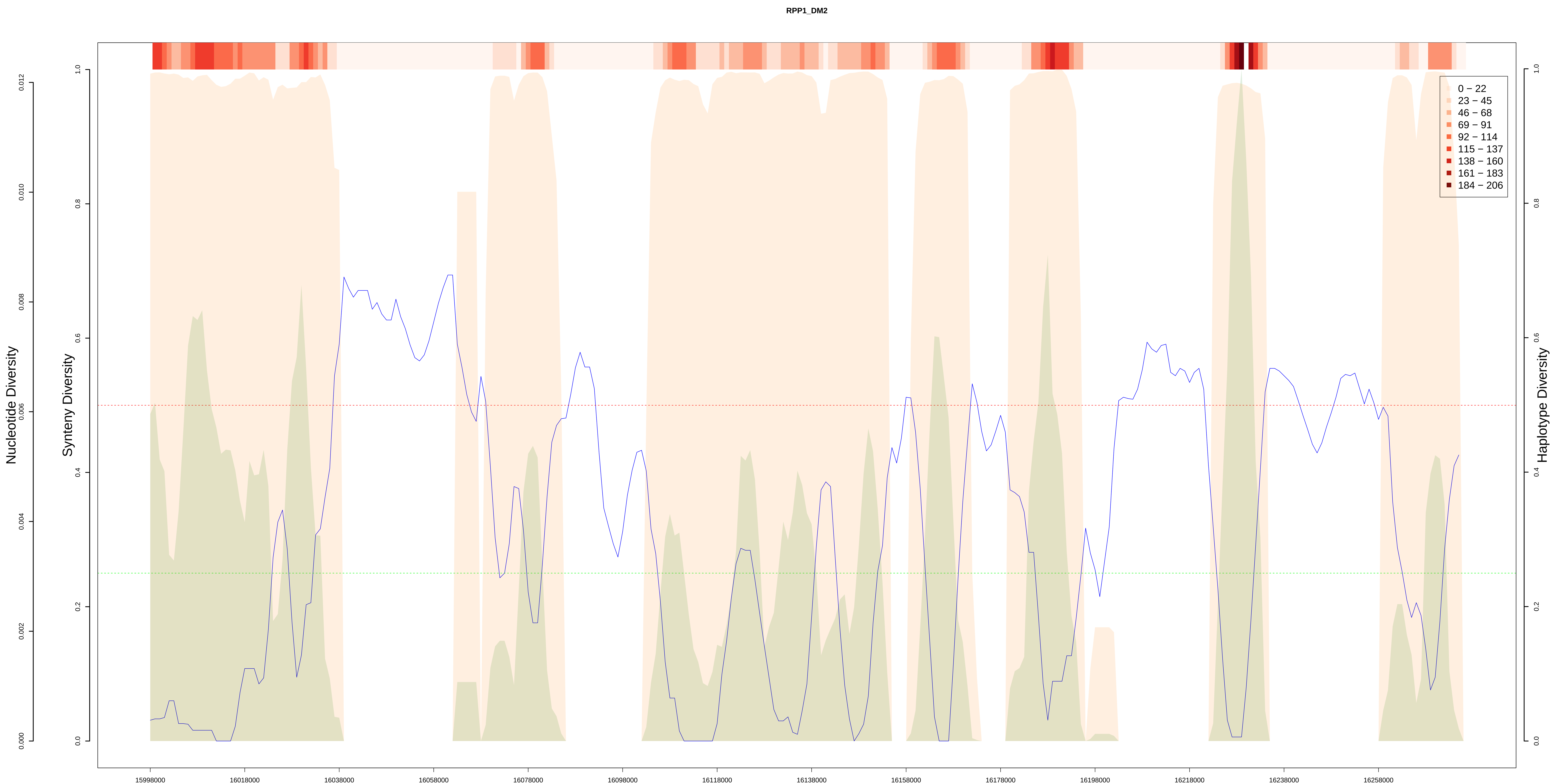

RPP1_DM2
Nucleotide Diversity
Synteny Diversity
Haplotype Diversity
0 – 22
23 – 45
46 – 68
69 – 91
92 – 114
115 – 137
138 – 160
161 – 183
184 – 206
15998000
16018000
16038000
16058000
16078000
16098000
16118000
16138000
16158000
16178000
16198000
16218000
16238000
16258000
0.000
0.002
0.004
0.006
0.008
0.010
0.012
0.0
0.2
0.4
0.6
0.8
1.0
