# Supplementary material for: Chromosome-level assemblies of multiple Arabidopsis genomes reveal hotspots of rearrangements with altered evolutionary dynamics
Source: Nat Commun. 2020 Feb 20;11:989. doi: 10.1038/s41467-020-14779-y (PMC7033125; doi:10.1038/s41467-020-14779-y)
Supplement: Supplementary file 8 — Source Data [file 41467_2020_14779_MOESM8_ESM.zip › Source_Data.Figure3.zip!/Source_Data.Figure3/3e/Col.vs.C24.nucmer.genes.pdf]

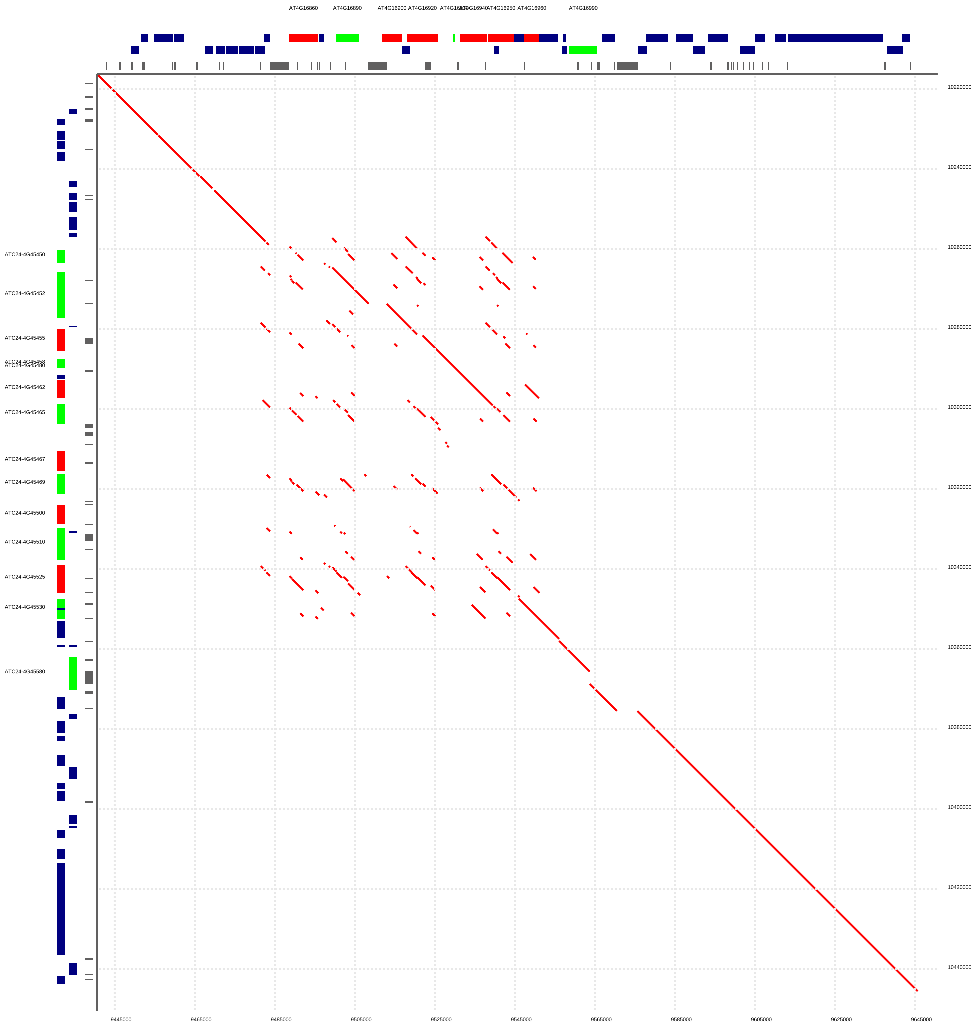
AT4G16860
AT4G16890
AT4G16900
AT4G16920
AT4G16940
AT4G16950
AT4G16960
AT4G16990
ATC24-4G45450
ATC24-4G45452
ATC24-4G45455
ATC24-4G45458
ATC24-4G45460
ATC24-4G45462
ATC24-4G45465
ATC24-4G45467
ATC24-4G45469
ATC24-4G45500
ATC24-4G45510
ATC24-4G45525
ATC24-4G45530
ATC24-4G45580
10220000
10240000
10260000
10280000
10300000
10320000
10340000
10360000
10380000
10400000
10420000
10440000
9445000
9465000
9485000
9505000
9525000
9545000
9565000
9585000
9605000
9625000
9645000
