# Supplementary material for: Chromosome-level assemblies of multiple Arabidopsis genomes reveal hotspots of rearrangements with altered evolutionary dynamics
Source: Nat Commun. 2020 Feb 20;11:989. doi: 10.1038/s41467-020-14779-y (PMC7033125; doi:10.1038/s41467-020-14779-y)
Supplement: Supplementary file 8 — Source Data [file 41467_2020_14779_MOESM8_ESM.zip › Source_Data.Figure3.zip!/Source_Data.Figure3/3d/RPP4_RPP5.win5kb.step1kb.popGenome.miss02.MAF005.pdf]

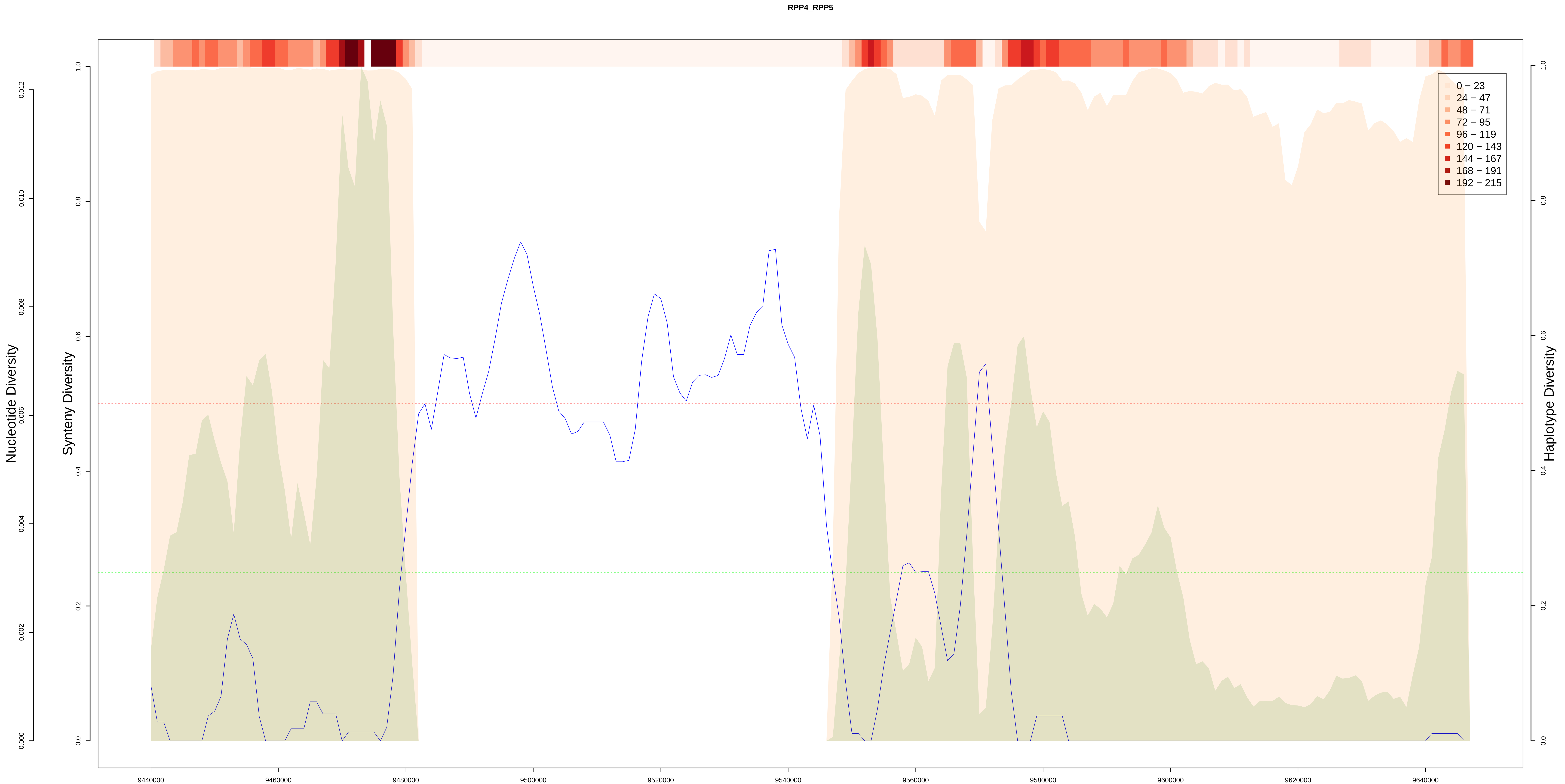
RPP4_RPP5
Nucleotide Diversity
Synteny Diversity
Haplotype Diversity
0 – 23
24 – 47
48 – 71
72 – 95
96 – 119
120 – 143
144 – 167
168 – 191
192 – 215
9440000
9460000
9480000
9500000
9520000
9540000
9560000
9580000
9600000
9620000
9640000
0.000
0.002
0.004
0.006
0.008
0.010
0.012
0.0
0.2
0.4
0.6
0.8
1.0
