# Supplementary material for: Chromosome-level assemblies of multiple Arabidopsis genomes reveal hotspots of rearrangements with altered evolutionary dynamics
Source: Nat Commun. 2020 Feb 20;11:989. doi: 10.1038/s41467-020-14779-y (PMC7033125; doi:10.1038/s41467-020-14779-y)
Supplement: Supplementary file 8 — Source Data [file 41467_2020_14779_MOESM8_ESM.zip › Source_Data.Figure4.zip!/Source_Data.Figure4/4b/Chr5_6.win5kb.step1kb.popGenome.miss02.MAF005.pdf]

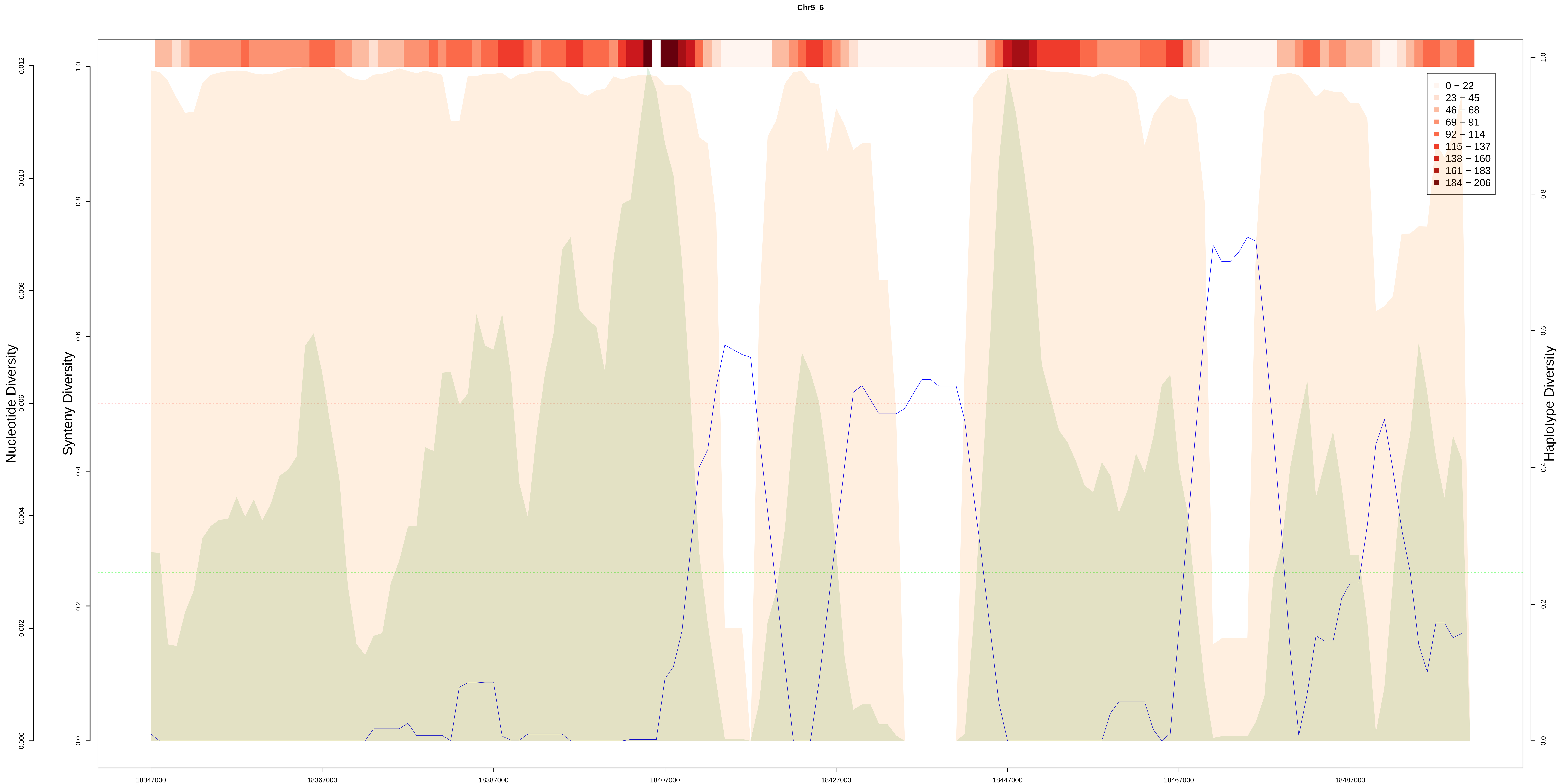

Chr5_6
Nucleotide Diversity
Synteny Diversity
Haplotype Diversity
0.000
0.002
0.004
0.006
0.008
0.010
0.012
0.0
0.2
0.4
0.6
0.8
1.0
0 – 22
23 – 45
46 – 68
69 – 91
92 – 114
115 – 137
138 – 160
161 – 183
184 – 206
18347000
18367000
18387000
18407000
18427000
18447000
18467000
18487000
