# Supplementary material for: Chromosome-level assemblies of multiple Arabidopsis genomes reveal hotspots of rearrangements with altered evolutionary dynamics
Source: Nat Commun. 2020 Feb 20;11:989. doi: 10.1038/s41467-020-14779-y (PMC7033125; doi:10.1038/s41467-020-14779-y)
Supplement: Supplementary file 8 — Source Data [file 41467_2020_14779_MOESM8_ESM.zip › Source_Data.Figure4.zip!/Source_Data.Figure4/4a/RPP7.AMPRIL.pdf]

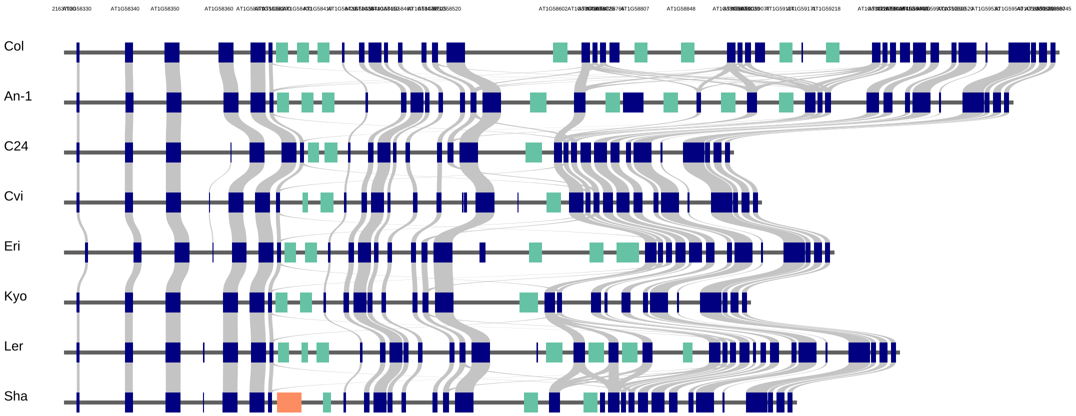

AT1G58340
AT1G58350
AT1G58360
AT1G58520
AT1G58807
AT1G58848
AT1G59218
Col
An-1
C24
Cvi
Eri
Kyo
Ler
Sha
