# Supplementary material for: Chromosome-level assemblies of multiple Arabidopsis genomes reveal hotspots of rearrangements with altered evolutionary dynamics
Source: Nat Commun. 2020 Feb 20;11:989. doi: 10.1038/s41467-020-14779-y (PMC7033125; doi:10.1038/s41467-020-14779-y)
Supplement: Supplementary file 8 — Source Data [file 41467_2020_14779_MOESM8_ESM.zip › Source_Data.Figure5.zip!/Source_Data.Figure5/5c/05-HDR.vs.syn.arm.MAF.hist.pdf]

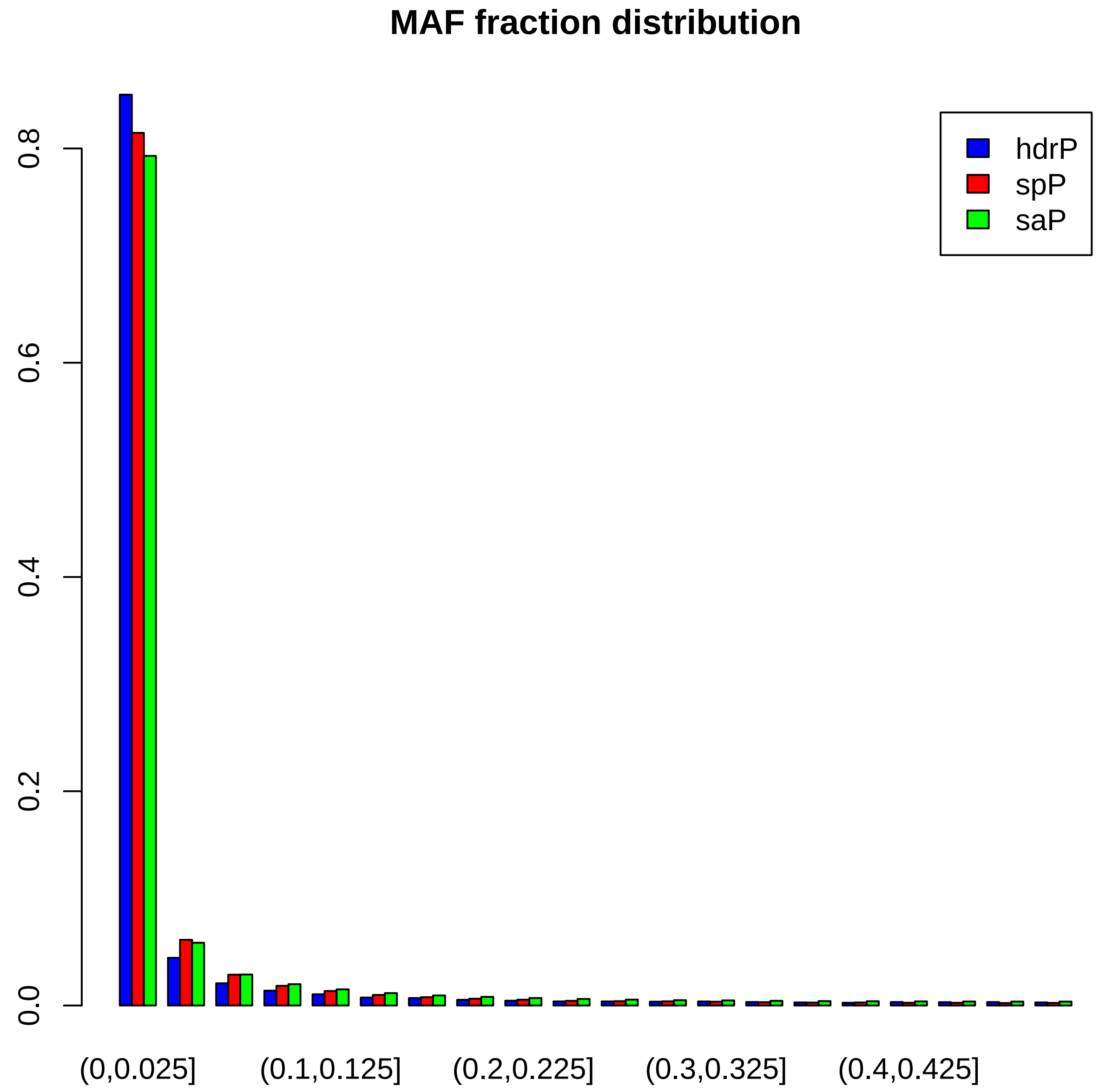
MAF fraction distribution
0.0
0.2
0.4
0.6
0.8
hdrP
spP
saP
(0,0.025]
(0.1,0.125]
(0.2,0.225]
(0.3,0.325]
(0.4,0.425]
