# Supplementary material for: Chromosome-level assemblies of multiple Arabidopsis genomes reveal hotspots of rearrangements with altered evolutionary dynamics
Source: Nat Commun. 2020 Feb 20;11:989. doi: 10.1038/s41467-020-14779-y (PMC7033125; doi:10.1038/s41467-020-14779-y)
Supplement: Supplementary file 8 — Source Data [file 41467_2020_14779_MOESM8_ESM.zip › Source_Data.SupplementaryFigure8.zip!/Source_Data.SupplementaryFigure8/Chr5_17440001_17446000.AMPRIL.pdf]

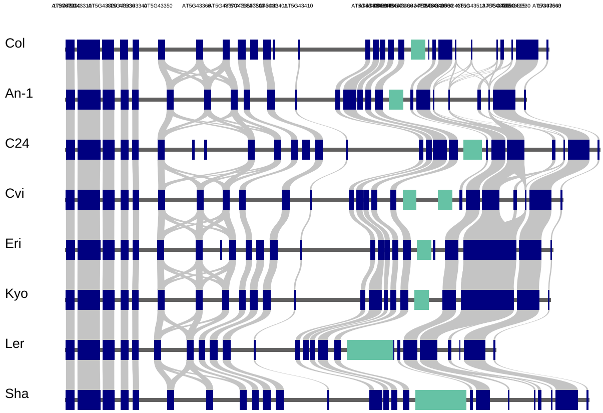
AT5G43350
AT5G43360
AT5G43410
Col
An-1
C24
Cvi
Eri
Kyo
Ler
Sha
