# Supplementary material for: Chromosome-level assemblies of multiple Arabidopsis genomes reveal hotspots of rearrangements with altered evolutionary dynamics
Source: Nat Commun. 2020 Feb 20;11:989. doi: 10.1038/s41467-020-14779-y (PMC7033125; doi:10.1038/s41467-020-14779-y)
Supplement: Supplementary file 8 — Source Data [file 41467_2020_14779_MOESM8_ESM.zip › Source_Data.SupplementaryFigure8.zip!/Source_Data.SupplementaryFigure8/Chr5_17440001_17446000.win5kb.step1kb.MAF005.pdf]

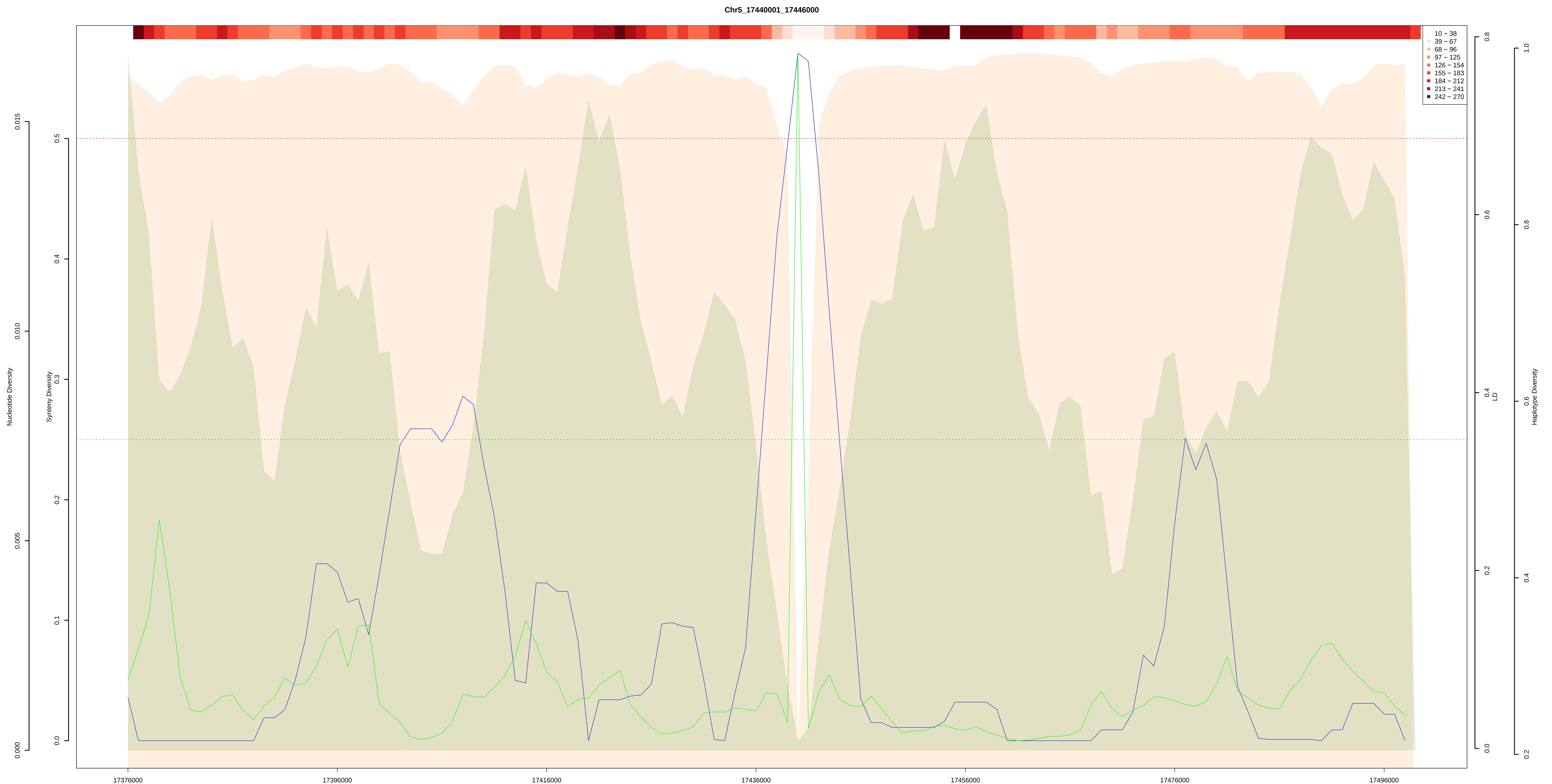

Chr5_17440001_17446000
10 − 38
39 − 67
68 − 96
97 − 125
126 − 154
155 − 183
184 − 212
213 − 241
242 − 270
Nucleotide Diversity
Synteny Diversity
LD
Haplotype Diversity
0.000
0.005
0.010
0.015
0.0
0.1
0.2
0.3
0.4
0.5
0.2
0.4
0.6
0.8
1.0
17376000
17396000
17416000
17436000
17456000
17476000
17496000
