# Supplementary material for: Chromosome-level assemblies of multiple Arabidopsis genomes reveal hotspots of rearrangements with altered evolutionary dynamics
Source: Nat Commun. 2020 Feb 20;11:989. doi: 10.1038/s41467-020-14779-y (PMC7033125; doi:10.1038/s41467-020-14779-y)
Supplement: Supplementary file 8 — Source Data [file 41467_2020_14779_MOESM8_ESM.zip › Source_Data.SupplementaryFigure12.zip!/Source_Data.SupplementaryFigure12/Chr5_24016001_24048000.win5kb.step1kb.MAF005.pdf]

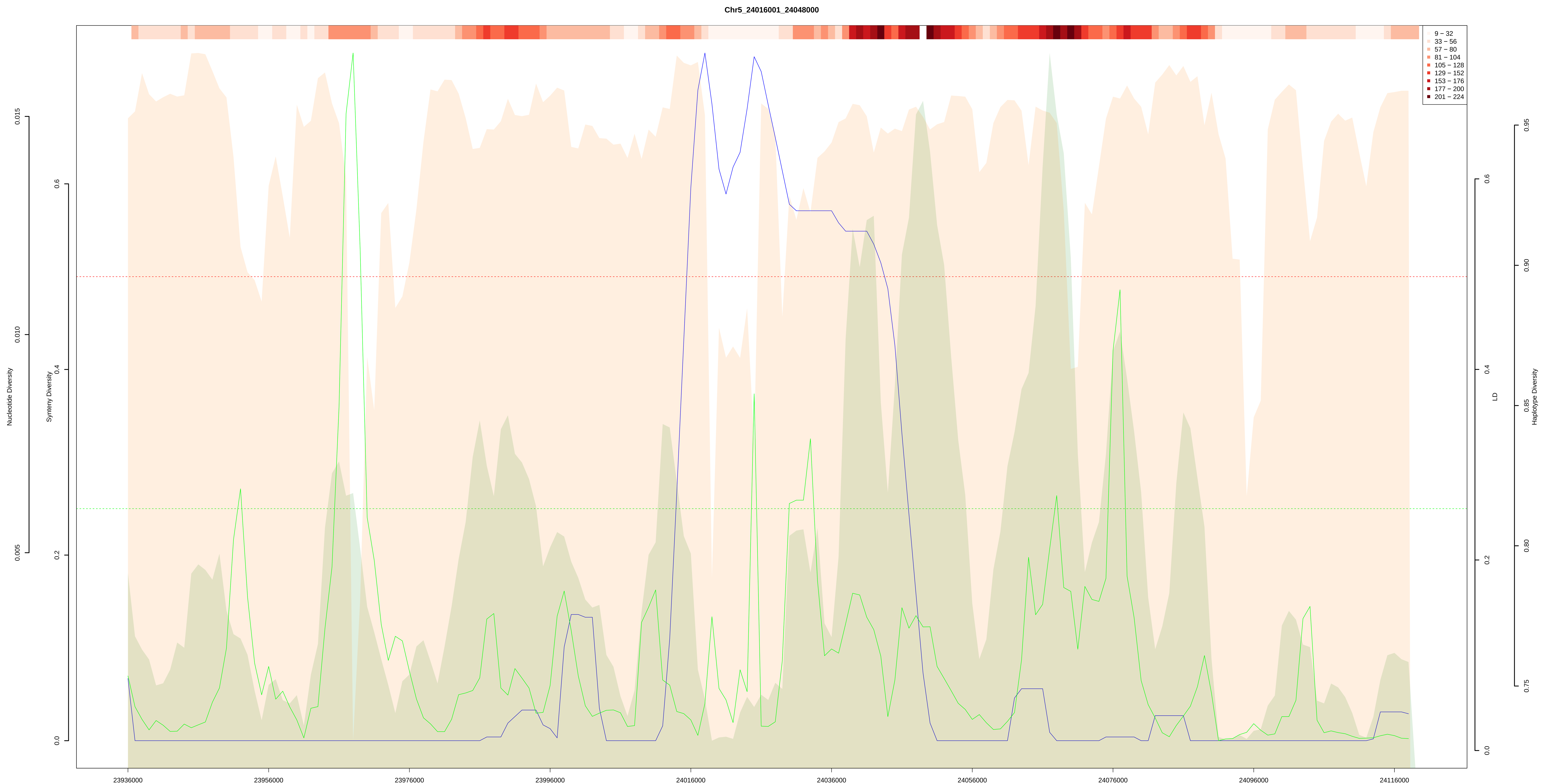

Chr5_24016001_24048000
Nucleotide Diversity
Synteny Diversity
LD
Haplotype Diversity
9 – 32
33 – 56
57 – 80
81 – 104
105 – 128
129 – 152
153 – 176
177 – 200
201 – 224
0.015
0.010
0.005
0.0
0.2
0.4
0.6
0.75
0.80
0.85
0.90
0.95
23936000
23956000
23976000
23996000
24016000
24036000
24056000
24076000
24096000
24116000
