# Supplementary material for: Chromosome-level assemblies of multiple Arabidopsis genomes reveal hotspots of rearrangements with altered evolutionary dynamics
Source: Nat Commun. 2020 Feb 20;11:989. doi: 10.1038/s41467-020-14779-y (PMC7033125; doi:10.1038/s41467-020-14779-y)
Supplement: Supplementary file 8 — Source Data [file 41467_2020_14779_MOESM8_ESM.zip › Source_Data.SupplementaryFigure10.zip!/Source_Data.SupplementaryFigure10/Chr3_18733001_18740000.win5kb.step1kb.MAF005.pdf]

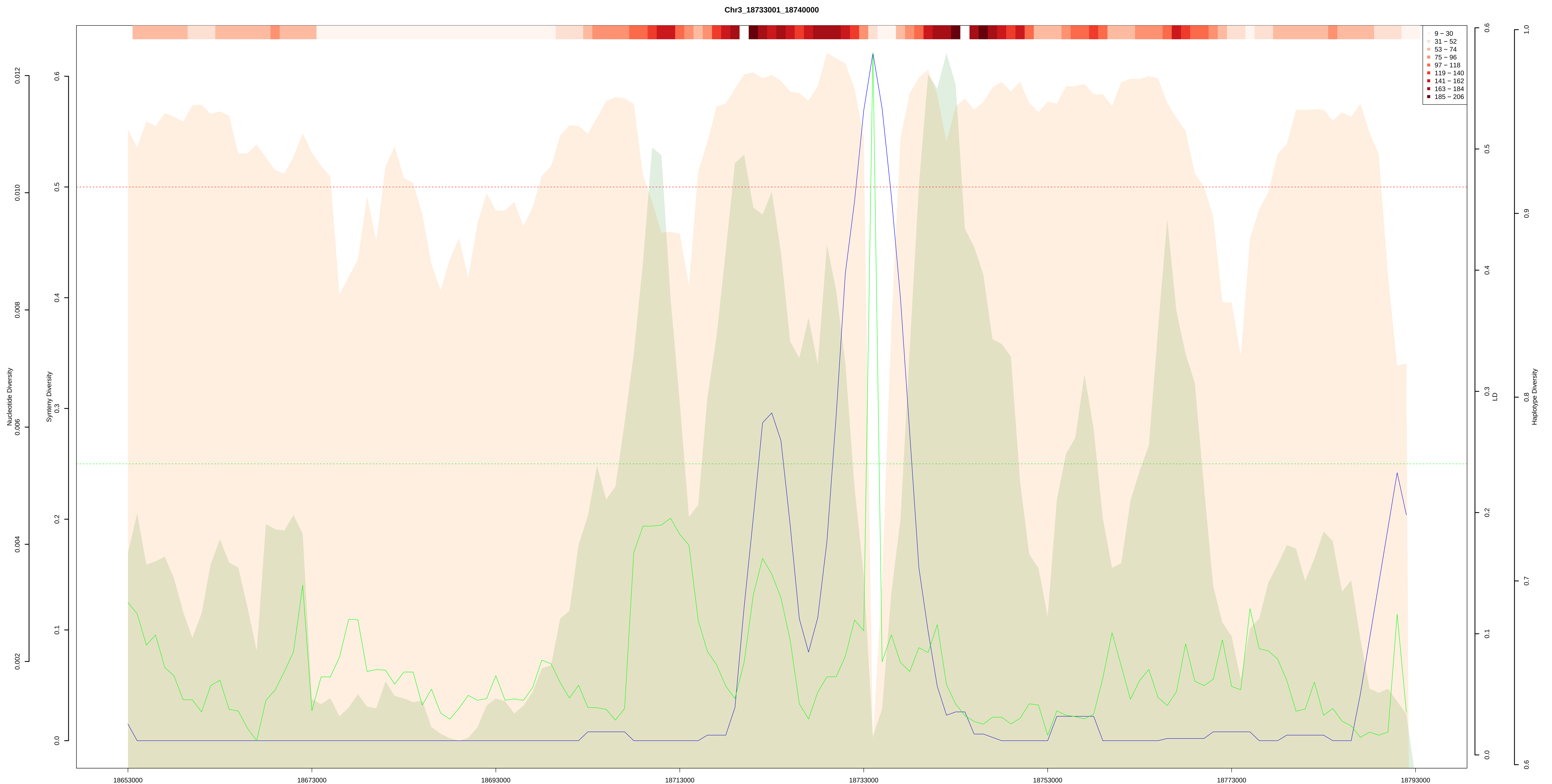
Chr3_18733001_18740000
Nucleotide Diversity
Synteny Diversity
LD
Haplotype Diversity
9 – 30
31 – 52
53 – 74
75 – 96
97 – 118
119 – 140
141 – 162
163 – 184
185 – 206
18653000
18673000
18693000
18713000
18733000
18753000
18773000
18793000
0.000
0.002
0.004
0.006
0.008
0.010
0.012
0.0
0.1
0.2
0.3
0.4
0.5
0.6
0.6
0.7
0.8
0.9
1.0
