# Supplementary material for: Chromosome-level assemblies of multiple Arabidopsis genomes reveal hotspots of rearrangements with altered evolutionary dynamics
Source: Nat Commun. 2020 Feb 20;11:989. doi: 10.1038/s41467-020-14779-y (PMC7033125; doi:10.1038/s41467-020-14779-y)
Supplement: Supplementary file 8 — Source Data [file 41467_2020_14779_MOESM8_ESM.zip › Source_Data.SupplementaryFigure11.zip!/Source_Data.SupplementaryFigure11/Chr4_8288001_8293000.win5kb.step1kb.MAF005.pdf]

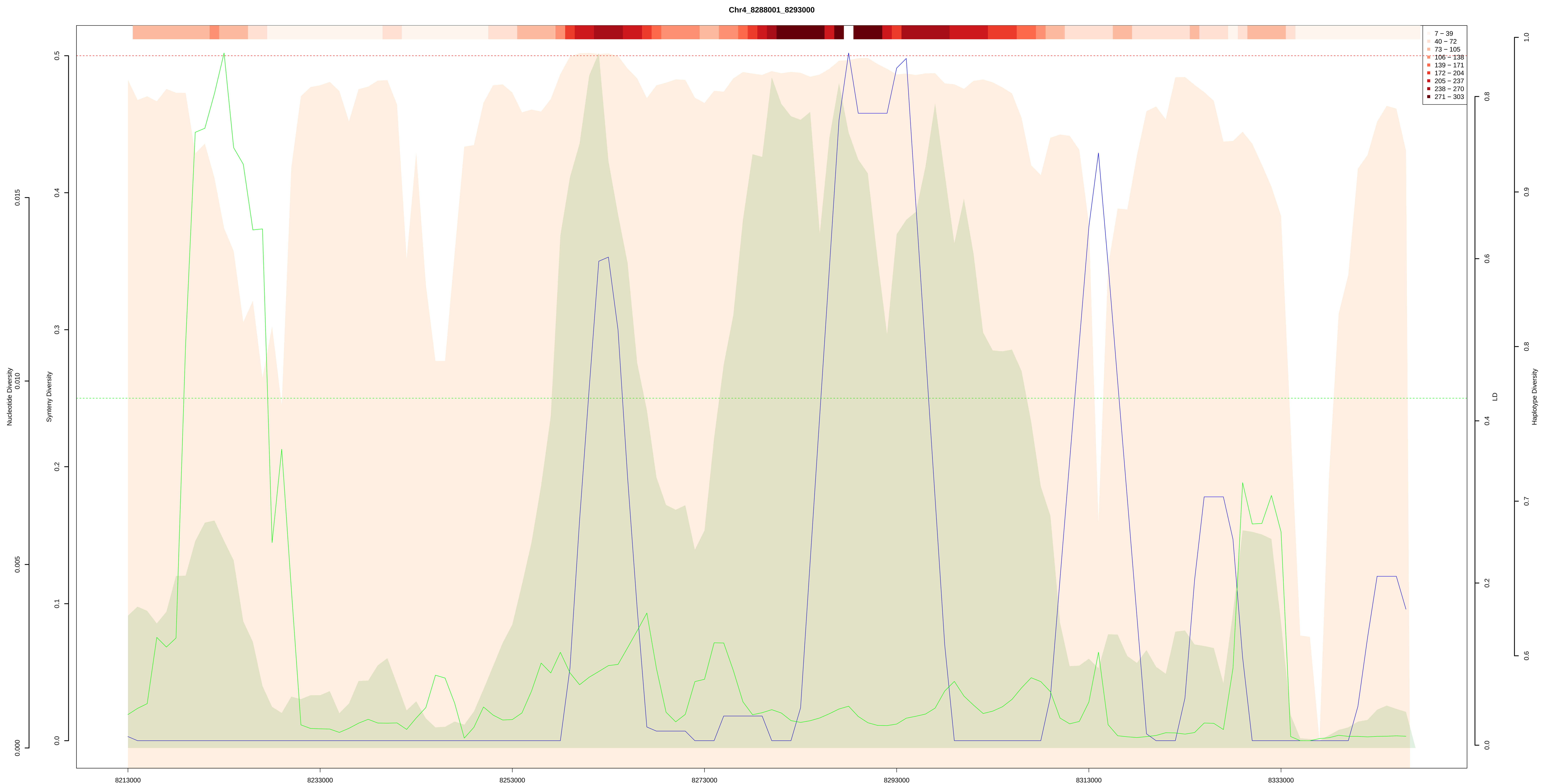

Chr4_8288001_8293000
7 – 39
40 – 72
73 – 105
106 – 138
139 – 171
172 – 204
205 – 237
238 – 270
271 – 303
Nucleotide Diversity
0.000
0.005
0.010
0.015
Synteny Diversity
0.0
0.1
0.2
0.3
0.4
0.5
LD
0.0
0.2
0.4
0.6
0.8
Haplotype Diversity
0.6
0.7
0.8
0.9
1.0
8213000
8233000
8253000
8273000
8293000
8313000
8333000
